# Tasquinimod (ABR-215050), a quinoline-3-carboxamide anti-angiogenic agent, modulates the expression of thrombospondin-1 in human prostate tumors

**DOI:** 10.1186/1476-4598-9-107

**Published:** 2010-05-17

**Authors:** Anders Olsson, Anders Björk, Johan Vallon-Christersson, John T Isaacs, Tomas Leanderson

**Affiliations:** 1 Active Biotech AB, Box 724, 22007 Lund, Sweden; 2 Department of Oncology, Clinical Sciences, and CREATE Health Strategic Center for Translational Cancer Research, Lund University, Lund, Sweden; 3 The Sidney Kimmel Comprehensive Cancer Center, The Johns Hopkins University School of Medicine, Baltimore, Maryland, USA; 4 Immunology Group, BMC D14, Lund University, 22184 Lund, Sweden

## Abstract

**Background:**

The orally active quinoline-3-carboxamide tasquinimod [ABR-215050; CAS number 254964-60-8), which currently is in a phase II-clinical trial in patients against metastatic prostate cancer, exhibits anti-tumor activity via inhibition of tumor angiogenesis in human and rodent tumors. To further explore the mode of action of tasquinimod, *in vitro *and *in vivo *experiments with gene microarray analysis were performed using LNCaP prostate tumor cells. The array data were validated by real-time semiquantitative reversed transcriptase polymerase chain reaction (sqRT-PCR) and protein expression techniques.

**Results:**

One of the most significant differentially expressed genes both *in vitro *and *in vivo *after exposure to tasquinimod, was thrombospondin-1 (TSP1). The up-regulation of TSP1 mRNA in LNCaP tumor cells both *in vitro *and *in vivo *correlated with an increased expression and extra cellular secretion of TSP1 protein. When nude mice bearing CWR-22RH human prostate tumors were treated with oral tasquinimod, there was a profound growth inhibition, associated with an up-regulation of TSP1 and a down- regulation of HIF-1 alpha protein, androgen receptor protein (AR) and glucose transporter-1 protein within the tumor tissue. Changes in TSP1 expression were paralleled by an anti-angiogenic response, as documented by decreased or unchanged tumor tissue levels of VEGF (a HIF-1 alpha down stream target) in the tumors from tasquinimod treated mice.

**Conclusions:**

We conclude that tasquinimod-induced up-regulation of TSP1 is part of a mechanism involving down-regulation of HIF1α and VEGF, which in turn leads to reduced angiogenesis via inhibition of the "angiogenic switch", that could explain tasquinimods therapeutic potential.

## Background

During the last decades, development of new cancer treatments that are capable of inhibiting tumor growth by inhibition of the blood supply has received great attention [1,2]. The quinoline compound tasquinimod [ABR-215050; CAS number 254964-60-8; 4-hydroxy-5-methoxy-N,1-dimethyl-2-oxo-N-[(4-trifluoromethyl) phenyl]-1,2-dihydroquinoline-3-carboxamide] has emerged as a candidate [3], by virtue of its pharmacological profile with anti-angiogenic and anti-tumor potency in experimental human prostate cancer models [4,5]. Thrombospondin-1 (TSP1) is a 450 kDa glycoprotein initially found in platelets, but also synthesized and secreted by many normal and transformed cells. TSP1 has been shown to be a potent natural inhibitor of tumor progression and metastases via inhibition of angiogenesis and migration or by activation of TGFβ (*for review see *[6-8]). Several mechanisms have been proposed for the anti-angiogenic properties of TSP1. For example, its type 1 repeat peptides (TSRs) [9] bind to and induce CD36-receptor signaling in endothelial cells and consequently lead to induction of apoptosis in endothelial tissue [10,11]. TSP1-induced signaling via CD47 and CD36 receptors expressed in endothelial cells also results in suppression of NO-dependent vascular pathways [12,13], and TSP1 acts as an apoptosis independent inhibitor of cell cycle progression [8]. Also, TSP1 inhibits neovascularization by interfering with FGF- and/or VEGF-induced angiogenesis via binding to, and blockage of, its receptors on the endothelial cell [14]. Furthermore, TSP1 negatively regulates the tissue levels of pro-angiogenic inducers (i.e. VEGF and FGF) and thereby shunts the "angiogenic switch" into an anti-angiogenic state [7,15]. Tissue level expressions of TSP1 are repressed by androgens in normal and cancer prostate tissue [16,17], and are down-regulated by several oncogenes including c-fos, c-jun, and Ras [18]. However, tumor suppressors such as PTEN, p53 and angiotensin II, but also PI3-kinase, β_1C _integrin, histone deacetylase (HDAC) inhibitors, and anti-cancer agents like dexrazoxane, up-regulate TSP1 levels [18-23]. Moreover, TSP1 modulates the expression of interleukin IL-6 and IL-10 by human monocytes [24], which both have impact on tumor vascularization. Lastly, TSP1 inhibits inflammation [25] and promotes the recruitment of M1-polarized macrophages (TAM:s) [26] that express high levels of iNOS (inducible NO synthetase) [27].

Many different approaches have been used to increase the systemic levels of TSP1 in order to inhibit angiogenesis and counteract cancer growth. Among these approaches are metronomic therapy (i.e. continuous low dose chemotherapy) [28], and systemic delivery of recombinant proteins [29] or synthetic peptides [13,30] with TSR sequences the most successful. In the present study we investigated the molecular changes that underlie the anti-tumor effects of tasquinimod in human prostate cancer cells (LNCaP). We observed elevated levels of TSP1 mRNA and protein expression in both cell cultures and tumor tissue after exposure to tasquinimod. Thus, we were able to identify a possible mechanism for the anti-tumor effect of tasquinimod, involving up-regulation of TSP1 and inhibition of the "angiogenic switch".

## Results

### Tasquinimods effects on gene expression in LNCaP cells analyzed with DNA microarray

**Figure 1:**
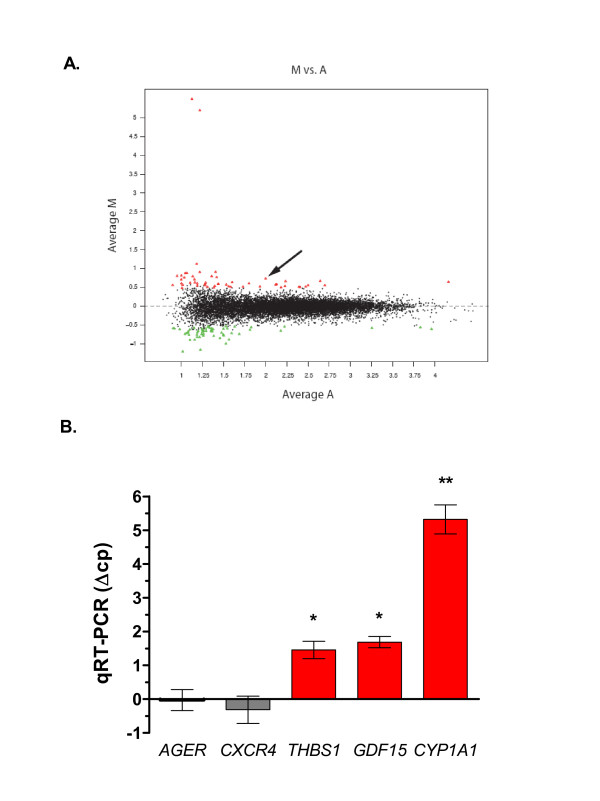
**Tasquinimod effects on gene expression in LNCaP cells analyzed with DNA microarray**. Altered gene expression induced by tasquinimod in LNCaP cell cultures. (A) Data plot of observed M ((M) = log_2_(Int1/Int2)) versus A (average intensity) from *in vitro *microarray experiment averaged over biological replicates (n = 4). *THBS1 *(arrow), GDF15 and CYP1A1 were significant outliers in all arrays analyzed (FDR < 10%; Table 1). (B) Validation of microarray data with semi-qRT-PCR confirmed the up-regulation of *THBS*, *GDF14 *and *CYP1A1 *(red bars; Δcp of 2.32 equals to a 5-fold change in mRNA expression). Expressed data represents the mean ± SD of at least two independent experiments. (*) p ≤ 0.05 and (**) p ≤ 0.01 compared with untreated control (Student's t-test).

Tasquinimod has been documented to have a robust and consistent anti-angiogenic activity *in vitro *at doses between 10-50 μM and *in vivo *at 1-10 mg/kg/day resulting in a potent anti-tumor effect in rodent and human prostate cancer xenografts tested in the same dose ranges [4,5]. Generated microarray data based on four separate biological replicates showed a drug-induced effect of 50 μM tasquinimod on gene expression in LNCaP cells when cultured *in vitro *for 24 h. Up- and down-regulation (FDR < 0.1; z-test) was noted for 107 genes (Additional file 1, Table S1). An *in vivo *experiment with LNCaP tumors inoculated (s.c.) into nude mice was also performed. Tumor bearing mice (n = 2) were exposed to ABR-215050 at 10 mg/kg for 24 h at day 14 and day 21 after inoculation before the tumors were excised. There was a similar gene expression pattern between the two 24 h time points, and for this reason day 14 and day 21 data were pooled (n ≤ 4, Additional file 2, Table S2). Table 1 lists a selected number of significantly up- or down-regulated genes after 24 h exposure to ABR-215050 (*in vivo *and/or *in vitro*). The presented genes were derived from significant clusters generated by a functional cluster analysis performed in the "DAVID and EASE" data base [31,32]. Briefly, the cluster analysis and pathway mapping revealed that many differentially expressed genes were involved in angiogenesis, cell cycle and apoptosis, migration, metabolism, signalling, DNA damage and oxidative stress (Table 1). Five of these genes, thrombospondin-1 (*THBS1*; TSP1), chemokine receptor *CXCR4*, cytochrome P450 1A1 (*CYP1A1*), receptor for advanced glycosylation endproduct (*AGER*; RAGE), and growth differentiation factor 15 (*GDF15*), were chosen for further validation using real time semi-quantitative RT-PCR. The expression data achieved by RT-PCR (Fig. 1B) were consistent with the microarray analysis data with a significant up-regulation of *THBS1*, *GDF15 *and *CYP1A1 *whereas *CXCR4 *and *AGER1 *did not change expression significantly (Fig. 1B and Table 1). Thus, the data achieved by microarray analysis after exposure to tasquinimod demonstrated a good agreement with semi-qRT-PCR as an independent validation method.

**Table 1:** Selected genes differentially expressed in LNCaP prostate tumor cells after exposure to tasquinimod for 24 h.

Functional groups* Gene name	**Accession #**^**†**^	**Gene**^**‡**^	Locus	*In vitro*	*In vivo*
***Angiogenesis/hypoxia***					
CD36 antigen (TSP1 receptor)	NM_001001547	CD36	948		**+**
Vascular endothelial growth factor	NM_001025366	VEGF	7442		**-**
Thrombospondin 1 (THBS1, TSP1)	NM_003246	THBS1	7057	**+**	+**
Endothelial PAS domain protein 1	NM_001430	EPAS1	2034		**+**
Neuropilin 2	NM_003872	NRP2	8828		**+**
Stabilin1	NM_015136	STAB1	23166		**+**
Angiopoietin 2	NM_001147	ANGPT2	285	**-**	**-**
Cadherin 13	NM_001257	CDH13	1012		**+**
Hypoxia-inducible protein 2	NM_013332	HIG2	29932		**-**
***Cell cycle/differentiation/senescence/apoptosis***					
Cyclin-depenent inhibitor kinase 1C	NM_000076	CDKN1C	1028		**-**
Adrenomedullin	NM_001124	ADM	133		**-**
Fibroblast growth factor	NM_033136	FGF1	2246		**+**
Advanced Glycosylation Endproduct Recept	NM_001136	AGER	177	**-**	
Leukocyte specific transcript protein 1	NM_007161	LST1	7941	**+**	
Forkhead box protein N4.	NM_213596	FOXN4	121643		**+**
Jun D proto-oncogene; AP-1 complex	NM_005354	JUND	3727		**-**
***Invasion/migration/adhesion/metastasis***					
Lysyloxidase preprotein	NM_002317	LOX	4015	**-**	**-**
Secreted phosphoprotein	NM_00582	SPP1	6996	**-**	
Chemokine (C-X-C motif) receptor 4	NM_003467	CXCR4	7852		**-**
Spleen tyrosine kinase	NM_003177	SYK	6850		**+**
Vimentin	NM_003380	VIM	7431		**+**
CD44 antigen isoform a	NM_000610	CD44	960		**+**
CD74 antigen	NM_004355	CD74	972		**+**
Insulin-like growth factor binding prot 3	NM_000598	IGFBP3	3486		**-**
S100 calcium-binding protein A6	NM_014624	S100A6	6277		**+**
S100 calcium binding protein P	NM_005980	S100P	6286		**+**
P8 protein (candidate of metastasis 1)	NM_012385	NUPR1	26471		**-**
Matrix metalloproteinase 14	NM_004995	MMP14	4323		**+**
Latent transforming GFB-binding prot	NM_032035	LTBP2	4053	**+**	
Insulin-like growth factor binding prot 7	NM_001553	IGFBP7	3490		**+**
***Stress response/metabolism/DNA repair***					
cytochrome P450, family 1, subfamily A	NM_000499	CYP1A1	1543	**+**	**+**
cytochrome P450, family 1, subfamily A	NM_000761	CYP1A2	1544	**+**	
growth differentiation factor 15 (NAG1)	NM_004864	GDF15	9518	**+**	
DNA-damage-ind. transcript 3 (GADD153)	NM_004083	DDIT3	1649	**+**	**-**
TCDD-inducible poly(ADP-ribose) polymerase	NM_015508	TIPARP	25976	**+**	**+**
Tumor protein p53 inducible nulear prot 2	NM_021202	TP53INP2	58476	**+**	
***Inflammatory response***					
Myeloperoxidase	NM_000250	MPO	4353	-	
Interleukin 8 receptor beta	NM_001557	IL8RB	3579		**-**
Interleukin 12 alpha chain	NM_000882	IL12A	3592	-	
Serpin peptidase inhibitor 1	NM_001085	SERPINA3	12	-	

* Functional classification by gene annotation enrichment analysis, gene clustering analysis and pathway mapping based on Gene Ontology data in DAVID Bioinformation database, ^**† **^Genebank accession number.

^**‡**^Presented genes were significantly up (+) or down (-) regulated (FDR < 10%) in ≥3 out of 4 biol replicates.

** Expressed in 2 out of 4 replicates

### TSP1 mRNA and protein induction by tasquinimod in vitro cell cultures of tumor cells

**Figure 2:**
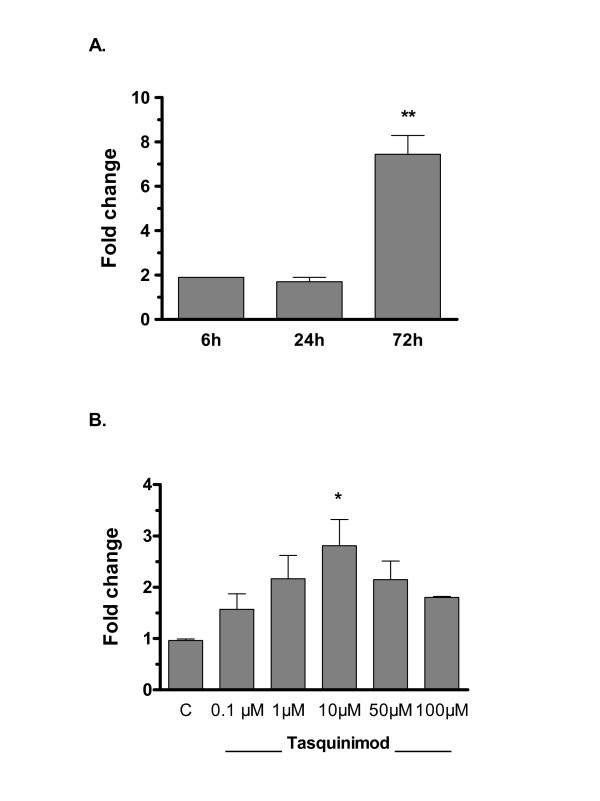
**TSP1 mRNA induction by tasquinimod in *in vitro *tumor cell cultures**. Tasquinimod-induced mRNA expression in LNCaP cells measured by real time semi-quantitative RT-PCR. (A) Time study after *in vitro *exposure with 10 μM tasquinimod, and (B) dose response with tasquinimod treatment between 0.1-100 μM for 72 h (p = 0.0128, ANOVA). Data expressed represents the mean ± SD of at least two independent experiments. (*) p ≤ 0.05 and (**) p ≤ 0.01 compared with untreated control (Bonferroni's multiple comparison test).

**Figure 3:**
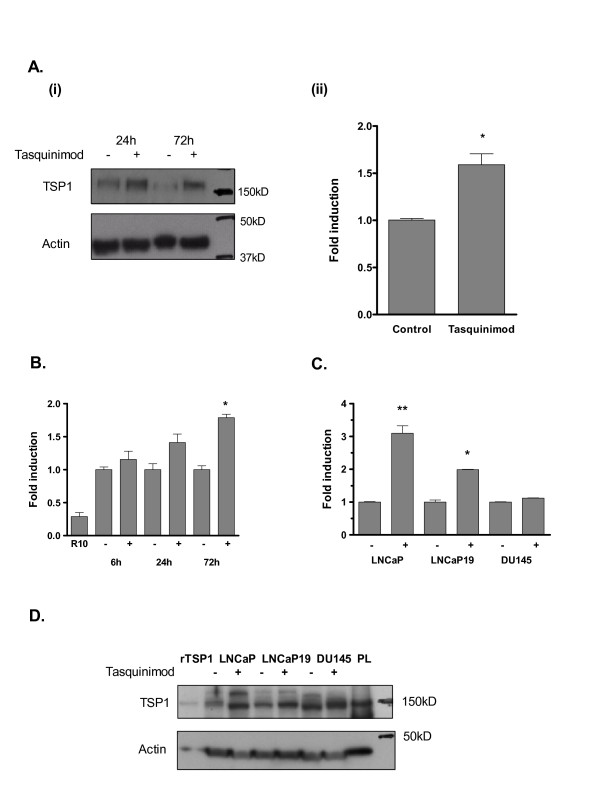
**TSP1 expression by LNCaP cells after tasquinimod exposure in *in vitro *cell cultures**. (A) Up-regulation of the TSP1 protein levels by tasquinimod (50 μM) in LNCaP cells was measured by western blot analysis (left panel (i)). Protein bands represent intact TSP1 at ~160 kD (monoclonal Ab11). TSP1 secreted into cell culture medium was measured with ELISA after 72 h (right panel (ii)). The culture medium levels of TSP1 were 50.8 ± 1.5 ng/ml for untreated cells and 80.6 ± 10.2 ng/ml for exposed cells, respectively (n = 3; p ≤ 0.05, ANOVA). (B) TSP1 secretion into cell culture medium after exposure of LNCaP cells to 10 μM tasquinimod (+) (p ≤ 0.01, ANOVA). TSP1 levels in untreated (-) cell culture medium were 22, 36.6 and 51.6 ng/ml after 6, 24 and 72 h incubation, respectively, and 6 ng/ml in the R10 medium. (C) Up-regulation of TSP1 mRNA levels occurred in the hormone independent prostate cancer cell line LNCaP19 but not in DU145, (-) untreated control and (+) 10 μM tasquinimod (p ≤ 0.0001; ANOVA). Presented data represent the mean ± SD of at least two independent experiments. (*) p ≤ 0.05 and (**) p ≤ 0.01 compared with untreated control (Bonferroni's multiple comparison test). (D) TSP1 protein levels measured by western blot analysis of prostate cancer cell lysates, (-) untreated control and (+) 10 μM tasquinimod for 72 h. PL indicates lysate prepared from human platelets and rTSP1 is recombinant TSP1.

Among the most significant gene differentially expressed both in cell cultures and in tumor tissue after exposure to tasquinimod, was TSP1 (*THBS1*; indicated by arrow in Fig. 1A). The up-regulation of TSP1 mRNA levels in LNCaP cells after exposure to tasquinimod was further investigated by semi-qRT-PCR. TSP1 mRNA induction was dependent both on dose and time. At 10 μM tasquinimod the TSP1 mRNA expression was elevated at 6 h and peaked after 72 h (Fig. 2A). Moreover, after 72 h exposure the TSP1 mRNA levels was already elevated at a dose of 1 μM tasquinimod (Fig 2B), indicating that tasquinimod-induced changes in TSP1 mRNA expression occurred in a dose range that has an documented anti-angiogenic and anti-tumor effect in *in vivo *xenograft models [5]. At higher dose levels (i.e. 50-100 μM) the mRNA levels declined at 72 h, indicating additional drug effects at these concentrations. The up-regulation of TSP1 mRNA in LNCaP cells by tasquinimod was manifested by an increased expression of TSP1 protein, as shown by western blot analysis of cell lysates prepared from cells cultured for 24 h and 72 h (Fig. 3A). Accompanied by increased intracellular TSP1 protein levels was also a statistically significant (p < 0.05) accumulation of extracellular TSP1 in the cell culture medium detected (Fig. 3A (ii)). The extracellular secretion of TSP1 was time dependent and could clearly be detected after 24 h exposure to tasquinimod at 10 μM (Fig. 3B). Also, TSP1 mRNA levels were induced by tasquinimod at 10 μM in the hormone insensitive cell line LNCaP19 but not in DU145 cells (Fig. 3C). The elevated mRNA levels was reflected in a minor increase of intracellular TSP1 protein levels in LNCaP19 cells expressed as a major band of intact protein around 150 kD (Fig. 3D). Taken together, the data obtained from *in vitro *exposure of human prostate cancer cells to tasquinimod demonstrate a drug effect resulting in increases of both TSP1 mRNA and protein expression.

### Up-regulation of TSP1 mRNA and protein levels in vivo in tumor tissue

**Figure 4:**
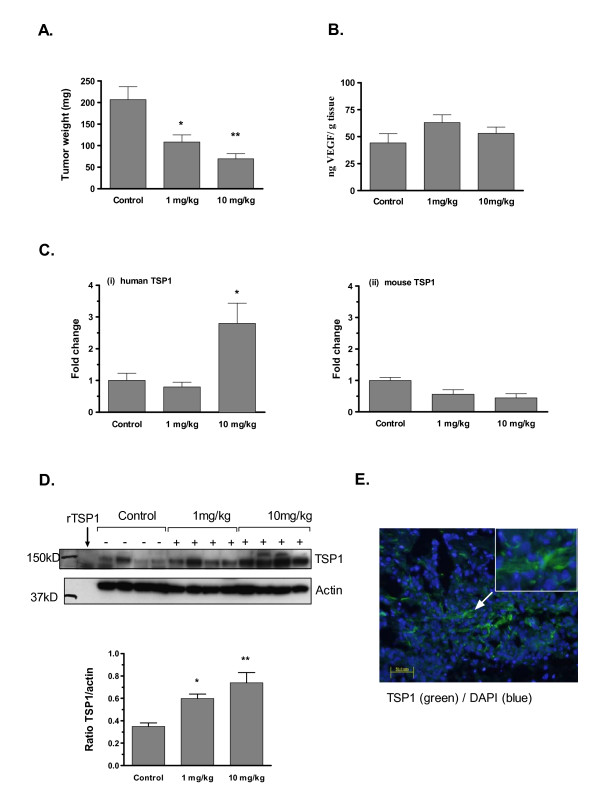
**Anti-tumor effect and up-regulation of tumor associated human TSP1 mRNA levels in tasquinimod treated LNCaP tumors**. Anti-tumor effect in nude mice carrying subcutaneous LNCaP tumors treated with tasquinimod (10 mg/kg/day) for three weeks. The treatment started 7 days after inoculation, and expressed data represent the mean tumor weight ± SD (n = 5; p = 0.0076, ANOVA). (B) VEGF levels were measured in processed tumor tissue by ELISA. (C) Up-regulation, monitored with real-time qRT-PCR, of tumor associated human TSP1 mRNA levels ((i); p = 0.0216, ANOVA) in LNCaP tumors. To distinguish between mRNA from human tumor cells and infiltrating mouse cells, TSP1 mRNA was analyzed using primers specific for human (i) or mouse (ii) sequences with the same probe set (Table 2). (D) Elevated protein levels of tumor-produced human TSP1 (rabbit polyclonal Ab8). Each lane represents a tumor sample from an individual animal. Calculated ratios between the major band of intact TSP1 (approximately at 150-160 kD) and actin in each lane show a significant (p = 0.0004, ANOVA) up-regulation of TSP1 tumor levels in exposed animals ((+); n = 4) compared to untreated controls (-). (*) p ≤ 0.05 and (**) p ≤ 0.01 (Bonferroni's multiple comparison test). (E) TSP1 expression (green) analyzed by IHC microscopy in LNCaP tumor tissue exposed *in vivo *to tasquinimod at 10 mg/kg/day (bar = 50 μm). TSP1 was mainly localized in the extra cellular matrix (inset). Blue shows DAPI staining.

Nude mice carrying subcutaneous LNCaP tumors were treated with tasquinimod for 3 weeks. Exposure to tasquinimod at 1 mg/kg/day and 10 mg/kg/day started on day 7 after inoculation. There was a statistically significant dose dependent reduction in tumor weight both at 1 mg/kg/day and 10 mg/kg/day compared to the untreated control group 28 days after inoculation (p < 0.001, ANOVA; Fig. 4A), illustrating the anti-tumor effect of tasquinimod. The tumor tissue levels of VEGF protein were not increased (Fig. 4B), whereas drug-induced changes in the tumor TSP1 mRNA expression at 10 mg/kg/day (p < 0.05; Fig. 4C (i)) and in protein levels at 1 and 10 mg/kg/day were significantly higher after 3 weeks exposure to tasquinimod (p < 0.001, ANOVA; Fig. 4D). The absence of mRNA induction at 1 mg/kg/day, however, may reflect the difficulty to detect smaller changes in mRNA levels after 3 weeks of continuous exposure at a relatively low dose and that mRNA expression and protein expression not always appear at the same levels in the tissue.

As a control, because the TSP1 antibody (rabbit polyclonal Ab8) cross-reacts with TSP1 of mouse origin, mRNA levels of mouse TSP1 was also measured, to assure that host derived TSP1 was not the source of increased TSP1 production in the treated tumors via infiltrating cells and blood (Fig. 4C(ii)). Immunoflouresence microscopy clearly showed TSP1 protein expression in LNCaP tumors (Fig. 4E). The most intense labeling was found in regions between zones of tumor growth and peripheral growth regions close to the tumor capsule ("the viable rim"). TSP1 was mainly localized in the extra cellular matrix in relation to blood vessels (Fig. 4E, inset). Thus, the elevated mRNA and protein levels of tumor derived TSP1 *in vivo *paralleled the observed changes in TSP1 mRNA- and protein expression *in vitro*.

### Tasquinimod blocks the angiogenic switch in CWR-22RH tumors

**Figure 5:**
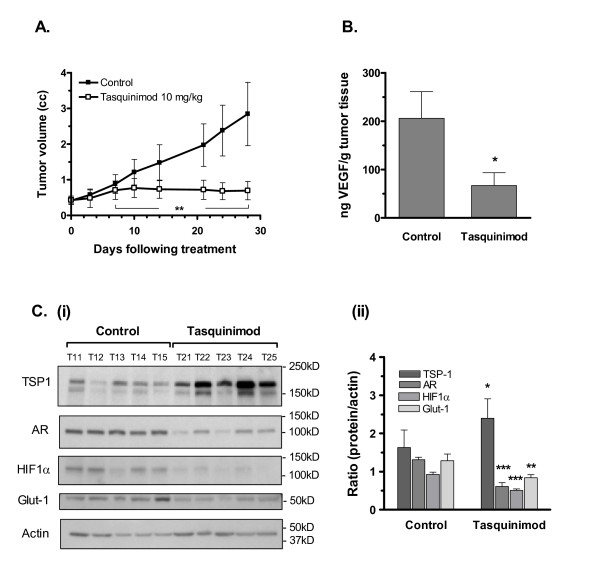
**Tasquinimod blocks the angiogenic switch in CWR-22RH tumors**. Inhibition of the "angiogenic switch" was illustrated in treated CWR-22RH tumors. (A) Tumor growth reduction of CWR-22RH human prostate tumors inoculated into nude mice after oral treatment with tasquinimod at 10 mg/kg/day, data points represent the average ± SD, (n = 5, (**) p = 0.002; Mann-Whitney U). (B) Reduced tumor levels of VEGF, a downstream HIF1α target gene. Bars represent the mean ± SD, n = 5 and (*) p < 0.05. (C) The up-regulation of TSP1 (mouse monoclonal Ab11) in tumor tissue excised and prepared as whole cell lysates (10,000 g supernatant) was accompanied with a down-regulation of the androgen receptor (AR), HIF1α, and Glut-1. (i) Each lane represents a tumor sample from an individual animal (#11 to #15 controls and #21 to #25 exposed to tasquinimod). Molecular weight markers for each blot are indicated. (ii) Calculated ratios between the major protein bands and actin for each lane show a statistical significant difference between exposed animals (#T21 - #T25) compared to untreated controls (#T11-#T15). (*) p ≤ 0.05, (**) p ≤ 0.01 and (***) p ≤ 0.001 (Bonferroni's multiple comparison test).

The term "angiogenic switch" denotes that in order for cancer to continuously grow, tumors must down-regulate natural angiogenesis inhibitors like TSP1, while coordinately up-regulating angiogenesis stimulators like VEGF [33]. When nude mice bearing CWR-22RH human prostate cancers were treated with oral tasquinimod, there was a profound growth inhibition (Fig. 5A; p < 0.01). Associated with this growth inhibition was a decreased tumor tissue level of VEGF (i.e. a down stream target of HIF1α) (Fig. 5B; p < 0.05). Likewise there was a significant up-regulation of TSP1 protein coupled with a down-regulation of HIF1α protein, androgen receptor protein (AR), and glucose transporter-1 protein (i.e. down stream target of HIF1α) within tasquinimod treated tumor tissues excised and prepared on day 28 (Fig. 5C; p < 0.05). Combined, these data clearly document that tasquinimod has inhibited the "angiogenic switch" within LNCaP and CWR-2RH xenografts.

## Discussion

In this report we have identified TSP1 as an important player in order to understand and explain the mechanism of the anti-angiogenic, and consequently the anti-tumor effect of tasquinimod treatment of prostate cancer. By using DNA microarray technique to monitor changes in mRNA expression after tasquinimod exposure, *in vitro *to cultured human LNCaP cells or *in vivo *to LNCaP tumors grown in nude mice, we were able to identify TSP1 as one potential candidate, among many other differentially expressed genes, for mediating the tasquinimod-induced anti-tumor activity. The increased expression of TSP1 mRNA was further validated by qRT-PCR and on protein level with western blot and histochemical analysis. Generally, there was a similar gene induction pattern on microarrays from tumor RNA after *in vivo *exposure. The good agreement between gene induction data achieved by microarray technique and RT-PCR was taken as evidence that the gene induction pattern observed on the microarrays were reliable and reflected an accurate picture of tasquinimod-induced gene expression. Despite the limited numbers of doses and time points, we were able to identify several interesting groups of functional clusters which may be involved in tasquinimods anti-tumor activity, such as angiogenesis, cell cycle regulation and migration (Table 1).

The nature of tasquinimod-induced increases in mRNA expression measured in tumor tissue after *in vivo *exposure is probably both of a direct and an indirect character. For example, TSP1, CYP1A1, and GDF15 mRNA induction are most likely reflecting a direct inducing effect on the tumor cells, because elevated mRNA levels were also achieved *in vitro *in cell cultures where no other cell types were present. On the contrary, genes involved in angiogenesis, cell cycle events or cell proliferation may be of an indirect or secondary nature and have a slower kinetic profile. With this in mind, up-regulation of for example the pro-angiogenic FGF1 in the LNCaP tumors were considered of minor importance compared to VEGF since we were not able to measure the FGF levels (by ELISA) in tumors or in cell culture medium from untreated cells or cells treated with tasquinimod (data not shown). TSP1 up-regulation in LNCaP cells involves different pathways including genes such as the *PI3K*, *Akt*, *PTEN*, *IGFBP3*, *p21 *(*Waf-1*) and *p53 *[19,22], and indeed some of these genes changed expression by tasquinimod exposure in our microarray experiments (Table 1).

In contrast, androgens and the androgen receptor (AR), which is expressed and functional in both LNCaP and CWR-22RH cells, have been reported to suppress TSP1 expression in tumor cells [34]. Colombel et al. showed that androgen ablation resulted in up-regulation of TSP1 which initially produced an anti-angiogenic response [17]. This therapeutic response was only temporary however, since the cancer cells in the resulting hypoxic environment eventually up-regulated the production of VEGF and thus became completely resistant to the enhanced TSP1 levels. In contrast to this rebound situation following androgen ablation, tasquinimod prevented such up-regulation of VEGF (Figs. 4B and 5B) and thus does not allow the tumor to overcome TSP1 anti-angiogenic effects. This may provide a mechanism that explains why tasquinimod treatment is synergistic when combined with androgen ablation [4]. Our data indicated a gradual difference in TSP1 induction and expression levels between androgen sensitive and refractive tumor cells. There was an evident induction of TSP1 expression at both mRNA and protein levels in the androgen sensitive tumor cells LNCaP and CWR-22RH, whereas there was less induction in LNCaP19, and no measurable induction above constitutive protein levels in the hormone refractive DU145 which lack the androgen receptor completely. Thus, tasquinimod exposure resulting in induction of TSP1 expression may involve effects on the AR and maybe also p53 activity, since cross-talk between these genes has been reported [35]. Besides transcriptional regulation, the anti-angiogenic activity by TSP1 requires proteolysis to liberate soluble anti-angiogenic N-terminal deletion fragments from extra-cellular matrix bound TSP1 and that tumor associated macrophages (TAM's) secrete ADAMTS1, which can perform this TSP1 fragmentation [36]. Once liberated, these soluble TSP1 N-terminal deletion fragments bind VEGF and FGF's in a way that prevents them from stimulating endothelial cells [37,38]. Such a process may require TAMs to attain tasquinimod's optimal activity *in vivo*. Recently it was reported that TSP1 modulates VEGF activity at the VEGF receptor level via its TSR interaction and co-clustering with CD36 and β1 integrins [39]. The documented anti-angiogenic and anti-tumor of up-regulated TSP1 in prostate cancers does however not always be the case for other types of cancers [40], indicating a dual effect of TSP1 on angiogenesis and tumor progression (*reviewed by *[41]).

The mode of action behind of the quinoline compounds anti-tumor properties has not been fully resolved, but several studies have shown that this group of compounds effect tumor angiogenesis [4,5], macrophage infiltration [42], cytokine production [43], and autoimmune/inflammatory disease [44]. Hence, the immuno-modularly properties of the quinolines indicate existence of an immunological component as a key player in quinoline compounds mode of action resulting in anti-tumor activity. This component is likely to be TAMs, and recently Martin-Manso et al. [26] reported that tumor-produced TSP1 promoted the recruitment of M1-polarized TAMs which stimulated reactive oxygen species (ROS)-mediated cytotoxicity of the endothelial cells and thereby caused inhibition of angiogenesis and tumor progression. By facilitating the infiltration of TAMs with a M1-phenotype (expressing high levels of iNOS and high production of NO) over TAMs of the proangiogenic M2-phenotype, it may be that tasquinimod-induced tumor levels of TSP1 change the balance between M1 and M2 cells. Thus, changes in such a critical ratio of NO to ROS generation would be able to destabilize the HIF1α protein even under hypoxic conditions [45]. This may explain tasquinimod's ability to provide an anti-angiogenic milieu by down-regulate HIF1α protein levels and its down stream targets, VEGF and Glut-1 proteins in human xenografts as they grow in nude mice (Fig. 5B &5C). Interesting, "M2-like" tumor infiltrating myeloid-derived suppressor cells (MDSCs) is a cell population with a phenotype similar to M2 macrophages expressing high levels of arginase-1 [46] and S100A9 protein [47]. The calcium and zink dependent protein S100A9 regulates the accumulation of tumor infiltrating MDSCs [48], and tasquinimod as a representative of quinoline compounds which recently were described as a strong binders to S100A9 [44], may have inhibited the MDSC accumulation and thereby prohibited MDSCs/M2 macrophages to act in a pro-angiogenic and pro-tumor fashion. Thus, the involvement of an additional mechanism could be possible, meaning that tasquinimod may act in LNCaP tumors both by inducing TSP1 production and TAM (M1) recruitment and at the same time inhibit the accumulation of MDSCs and prevent M2 macrophage action via direct interference with the S100A9 protein in immature myeloid cells. This possibility will be the subject of future studies of the mechanism of action of tasquinimod.

## Conclusions

We conclude that tasquinimod-induced up-regulation of TSP1 is part of a mechanism involving down-regulation of HIF1α and VEGF, which in turn leads to reduced angiogenesis via inhibition of the "angiogenic switch", that could explain tasquinimod's therapeutic potential

## Materials and methods

### Cell culture and materials

Tasquinimod was synthesized at Active Biotech Research AB (Lund, Sweden), as previously described [49]. In this study tasquinimod was used *in vitro *at doses between 0.1-100 μM and at 1 and 10 mg/kg/day *in vivo*, based upon pharmacokinetic studies [5] where daily oral dosing with 1-30 mg/kg/day produced serum levels of tasquinimod in the 1-4 μM range. Two human prostate cancer cell lines, CWR-22RH and LNCaP (ATCC) are both androgen independent, but remain sensitive to androgen stimulation of growth, express PSA and a mutated androgen receptor [4]. The hormone independent cell lines LNCaP19 [50] and DU145 were also tested for TSP1 induction after *in vitro *exposure to tasquinimod. CWR-22RH, LNCaP and DU145 were grown in RPMI Medium 1640 containing 10% FCS and L-Glutamine mixture, while LNCAP19 was cultured in RPMI-medium with 10% hormone free (RDCC) FCS.

### Animals

Nude BALB/c mice were used for subcutaneous implantation of human prostate tumor cells LNCaP and CWR-22RH [4]. All animal experiments were conducted in accordance with the Bioethics Committee guidelines in Lund, Sweden. Tumor growth was measured with a microcaliper twice a week throughout the experiment, and the final tumor burden was measured by weight on the day of termination of the experiment. Distribution of tasquinimod at 1 mg/kg/day and 10 mg/kg/day (administered orally via the drinking water) started on day 7 after inoculation.

### RNA preparation

Tissue samples *in vitro *or *in vivo *were collected and stored at -70°C in Trizol^® ^(Invitrogen). The total RNA was isolated by extracting the Trizol^® ^samples with chloroform followed by separation and purification using a modified protocol for the RNeasy RNA extraction Kit (Qiagen). 1 μl RNAse inhibitor mix was added per 50 μl total RNA before treatment with DNAse for 20 min (DNA-free™; Ambion, Austin TX). The RNA concentration and purity was quality controlled by analysis with a Bioanalyser.

### Microarray experiments

LNCaP cells were exposed *in vitro *to 50 μM tasquinimod for 24 h before harvest and RNA extraction. Tumor bearing mice (LNCaP inoculated in nude mice) were treated with tasquinimod at 10 mg/kg (ad.lib.) and the tumors of each of the 2 different treatment groups were excised after 24 h of treatment (start day 14 or day 21 after inoculation) and total RNA was isolated. Untreated samples were grown and harvested in parallel and used as controls for microarray experiments. The RNA from treated and control samples were differentially labeled with fluorescent dye (Cy3 & Cy5) and hybridized on human oligo-arrays as described [51]. All hybridizations were replicated using dye-swap for the flourecent dyes, and data from image-analysis were loaded into a local installation of BioArray Software Environment (BASE) [52] for preprocessing and normalization. Spots from dye-swap hybridization were merged using geometric mean of ratios requiring presence in both replicates. For spots, log ratio (M) was calculated as log_2_(Int1/Int2) (Int1 = treated sample intensity and Int2 = control intensity) and average intensity (A) was calculated as log_10_(Int1*Int2)/2. Normalization was performed by applying Lowess on M values stratified into 8 separate groups defined spatially by pin-tip (8 neighboring pin-tips per group). Probe replicates were merged and a probe presence in 3 of 4 biological replicates was required for inclusion in further analysis. All microarray data will be made available in NCBI's Gene Expression Omnibus (GEO) accessible through GEO Series entry GSE17031.

### Real-time semi-quantitative Reversed Transcriptase-PCR (semi-qRT-PCR)

Total RNA was reversed transcribed using the "Transcriptor First Strand cDNA Synthesis Kit" (Roche) and anchored oligo-(dT)_18 _primers. PCR primers and fluorogenic probes (with an 640 tag) for the human "house-keeping" gene hGAPDH and the five genes to validate, *THBS1*, *CXCR4*, *CYP1A1*, *GDF15 *and *AGER *were designed, synthesized and purified by TIB MOLBIOL (Berlin, Germany) (Table 2). Real Time PCR was performed using the "LightCycler FastStart DNAMaster^plus ^Hybidization Probes" kit (Roche). Cycling parameters were as follows: Denaturation step for 10 min at 94°C//Amplification step (40-50 cycles) was 10 s at 95°C _ 10 s at 56°C _ 20 s at 72°C//30 s at 33°C, the transition rate was 20°C/s for all steps except for 72°C to 90°C which was 10°C/s. To measure up- or down-regulation of cellular mRNA levels, CP-values (i.e. GAPDH - tested gene) were calculated from at least three different analysis using varied cDNA concentrations.

**Table 2:** Primer and probe sets used for RT-PCR and semi-quantitative real time PCR*.

***Target gene***	***Primer*** ***Sequence (5' => 3')***	***Probe***^**†**^ ***Sequence (5' => 3')***	***Amplicon length (bp)***
***hu GAPDH***	FW: gAAggTgAAggTCggAgTC Rev: gAAgATggTgATgggATTTC	AggggTCATTgATggCAACAATATCCA-**FL** **LC**:640-TTTACCAgAgTTAAAAgCAgCCCTggTg	226
			
***hu TSP-1***	FW: CTggACTCgCTgTAggTTA Rev: CCCTgTggTggAgTTTAC	AgTCATCgTCCCTTTCggTg-**FL** **LC**: 640-TgATgAAgAAggTgCCACTgAAgT	233
			
***hu CXCR4***	FW: CgAggAAATgggCTCAg Rev: gggAAgCgTgATgACAAA	TTTTATTgAAATTAgCATTTTCTTCACggA-**FL** **LC**:640-ACAgggTTCCTTCATggAgTCATAgTCC	273
			
***hu CYP1A1***	FW: gAgCTgggTTTgACACAgTC Rev: ggATgTAAAAgCCTTTCAAACT	gTCggAAggTCTCCAggATgAAg-**FL** **LC**: 640-CCTCCATATAgggCAgATgggATCTg	258
			
***hu GDF15***	FW:gAAgACTCCAgATTCCgAgAgTT Rev:gATCCCAgCCgCACTTCT	gATTCgAACACCgACCTCgTCCC-**FL** **LC**: 640-gCCCCTgCAgTCCggATACTCAC	146
			
***hu AGER***	FW: TCTgCCTCTgAACTCACgg Rev: CCTTCACAgATACTCCCTTCTCATT	CAgggACTCTTAgCTggCACTTgg--**FL** **LC**: 640-TgggAAAgCCCCTggTgCCT	139
			
***huTSP-1***^**‡**^	FW: AACggAgTTCAgTACAgAAAT Rev: TTCCATTgCCACAgCTC	CAgAACTCRgTTACCATCTgCAAAAAgg-**FL** **LC**: 640-gTCCTgYCCCATCATgCCCTgC	235
			
***mTsp-1***^**‡**^	FW: AgAgAACAgAgAgCTggTCAg Rev: ATCTgTTgTTgAggCTgTCA		329
			
***mPbgd***	FW:TTgTACCCTggCATACAgTTTgA Rev: gTTCCCACggCACTTTTC	TgAAggATgTgCCTACCATACTACCTCCT-**FL** **LC**: 640-gCTTTACTATTggAgCCATCTgCAAAC	247

* Designed, synthesized and purified by TIB MOLBIOL (Berlin, Germany)

^† ^Probes designated LC contain the acceptor dye LigtCycler Red 640 covalently attached at the 5'-end, and the FL probes contain the donor dye Fluorescein at the 3'-end.

^‡ ^Specific mouse- and human *TSP1 *primers sharing the same LC/FL probe sequence for *TSP1*.

### Western blotting

SDS-/PAGE and western blot analysis was performed on the 10,000 g supernatant fraction of total soluble proteins prepared from cell pellets or from frozen tumor tissue (100 μg). Briefly, frozen tumor tissue was weighed and diced into small pieces. Ice cold lysis buffer (20 mM Tris HCl pH 7.4 with 150 mM NaCl, 1 mM EDTA, 0.5% NP-40) 0.5% sodium deoxycholate, 1 mM DTT, protease inhibitor cocktail (Roche), 1.5 mM sodium vanadate, 1 μM cystine and 80 mM sodium glycerophosphate) was added, homogenized, and centrifuged at 10,000 g for 15 minutes. 10 μg protein was loaded in each lane on a 4-12% Bis-Tris gel, the protein transferred to a PVDF transfer membrane (Immobilon-P, Millipore), and blocked overnight in 5% dry fat-free milk in TBST. Incubation with anti-hTSP1 Ab11 (clones D4.6 + A6.1 + MBC200.1) or Ab8 (rabbit polyclonal; Lab Vision, NeoMarkers CA, USA) was performed using mouse anti-human-actin as an internal control (Sigma). Both antibodies showed cross-reactivity to mouse derived TSP1 and the detection patterns were similar for both antibodies (i.e. Ab8 and Ab11). Recombinant human TSP1 protein (140 kD, ProSpec-Tany TechnoGene LTD, Israel) was loaded at 3 ng and anti-bodies against androgen receptor (AR; rabbit polyclonal, (N-20) Santa Cruz), HIF1α (mouse monoclonal from BD) and Glut1 (rabbit polyclonal, (H-43) Santa Cruz) were all used at 1:500 dilution.

As secondary detection antibodies, (HRP)-conjugated anti-rabbit antibody or anti-mouse antibodies (Amersham) were used, followed by detection with the chemoluminiscence ECL-Plus reagent (Amersham, UK), developed on ECL Hyperfilm (GE Healthcare, UK), captured and analyzed using a Kodak Digital Science™ Image Station 440F.

### Elisa measurements

VEGF content in tumor tissue and TSP1 levels in cell culture medium were measured by ELISA (VEGF, Biosurce, Belgium; #KHG0112, and TSP1, Quantikine^® ^R&D Systems Europe Ltd, UK; #DTSP10) according to the manufacturer's protocol.

### Immuno histoflouresence (IHF) microscopy

Tumor tissue, were fixed in 4% paraformaldehyde (PBS), immersed in 15% and 25% sucrose/PBS, tissues were mounted in Tissue-Tek (Sakura Finetek Europe, Netherland) and immediately frozen on carbon dioxide ice. Cryosections were cut (10 μm) and two sections were collected per slide, comprising at least two levels of the tumor. General tissue morphology was visualized by hematoxylin and eosin staining. For immunohisto-fluorescence (IHF) labeling of frozen tissue sections was the anti-human TSP1 antibody (Ab8).IHF labeling was accompanied by nuclear staining with DAPI (Molecular Probes, USA).

### Statistical analysis

For microarray experiments, genes were selected for up- or down-regulated transcript expression after exposure to tasquinimod compared to non-exposed controls. Using independent biological replicates the probability of observed mean M was calculated for each reporter using a BASE plug-in implementation of one-class Z-test [52] with the null hypothesis that the reporter is non-differentially expressed. The null distribution was derived from pooled standard deviation and mean for the biological replicates. Obtained probabilities (P) were used to rank reporters and to calculate expected number of reporters (expected) for each rank. False discovery rate (FDR) was estimated by comparing the expected number of reporters and rank, i.e., expected/rank. To select differentially expressed reporters a cut-off was set at false discovery rate 10% (FDR < 0.10) [52].

The statistical significance of difference between experimental conditions was determined using One-way ANOVA analysis (p ≤ 0.05 based on F values) with two-tailed Student's t-test with Bonferroni's correction for comparison of multiple groups, (*) p ≤ 0.05 and (**) p ≤ 0.01.

## Competing interests

AO, AB and TL are employees of Active Biotech AB that is a company developing quinolines for commercial purposes. JVC and JTI declare that they have no competing interests.

## Authors' contributions

AO, AB and TL initiated and planned the project. AO performed the experimental design, analyzed the microarray data, performed pathway analyses, designed primers, carried out QPCR, ELISA and western blots, drafted and wrote the manuscript. TL participated in the set up of the initial study, the experimental design, carried out the overall responsibility of the research performed and revised the manuscript critically. JVC planned, processed and analyzed the microarray data, performed statistical analyses, contributed to the writing of the manuscript and made the microarray data available in NCBI's Gene Expression Omnibus. JTI planned, performed and analyzed all the experiments associated with the CWR-22RH tumor model and changes in the angiogenic switch. All authors read and approved the final manuscript.

## Supplementary Material

Additional file 1 **Table S1 - Genes up- and down regulated at FDR < 0.1 (10%) after *in vitro *exposure to Tasquinimod for 24 h**. The indicated column headings (**bold**) are explained as follows (also indicated as foot notes): (+) indicates **up**-regulation in treated vs untreated and (-) indicates **down**-regulation in treated versus untreated*, Absolute **fold change **between untreated and treated cells**†**, **Average M **of biological replicates**‡**, **n **= number of biological replicates**, **P **= probability of obtaining the observed average M (Z-test) **††**, **Rank **= ranked genes based on obtained probabilities (i.e., the reporter with lowest P will have rank 1) **‡‡**, **Expected **= expected number of reporters calculated as probability times total number of reporters ***, and **FDR **= number of expected number of reporters divided by observed number of reporters (rank) **‡‡‡**. Click here for file

Additional file 2 **Table S2 - Genes up- or down-regulated at FDR < 0.1 (10%) after *in vivo *exposure to Tasquinimod for 24 h at 10 mg/kg**. The indicated column headings (**bold**) are explained as follows (also indicated as foot notes): (+) indicates **up**-regulation in treated vs untreated and (-) indicates **down**-regulation in treated versus untreated*, Absolute **fold change **between untreated and treated cells**†**, **Average M **of biological replicates**‡**, **n **= number of biological replicates**, **P **= probability of obtaining the observed average M (Z-test) **††**, **Rank **= ranked genes based on obtained probabilities (i.e., the reporter with lowest P will have rank 1) **‡‡**, **Expected **= expected number of reporters calculated as probability times total number of reporters ***, and **FDR **= number of expected number of reporters divided by observed number of reporters (rank) **‡‡‡**. Click here for file
