# Supplementary material for: Tasquinimod (ABR-215050), a quinoline-3-carboxamide anti-angiogenic agent, modulates the expression of thrombospondin-1 in human prostate tumors
Source: Mol Cancer. 2010 May 17;9:107. doi: 10.1186/1476-4598-9-107 (PMC2885345; doi:10.1186/1476-4598-9-107)
Supplement: Additional file 1 — Table S1 - Genes up- and down regulated at FDR < 0.1 (10%) after in vitro exposure to Tasquinimod for 24 h. The indicated column headings (bold) are explained as follows (also indicated as foot notes): (+) indicates up-regulation in treated vs untreated and (-) indicates down-regulation in treated versus untreated*, Absolute fold change between untreated and treated cells†, Average M of biological replicates‡, n = number of biological replicates**, P = probability of obtaining the observed average M (Z-test) ††, Rank = ranked genes based on obtained probabilities (i.e., the reporter with lowest P will have rank 1) ‡‡, Expected = expected number of reporters calculated as probability times total number of reporters ***, and FDR = number of expected number of reporters divided by observed number of reporters (rank) ‡‡‡. [file 1476-4598-9-107-S1.PDF]

Additional File 1, Table S1.

Genes up- and down regulated at FDR< 0.1 (10%) after 24h in vitro exposure to Tasquinimod

reporterId	geneSymbol	up/down*	Fold change†	Average M‡	n**	P††	Rank‡‡	Expected***	FDR‡‡‡
H200005807	CYP1A1	+	45.12	5.50	4	0	1	0	0
H200005712	MOCS2	+	36.59	5.19	4	0	2	0	0
H300020746	C9orf117	-	2.30	-1.20	3	0	3	0.000002	0.000001
H200020844	ODZ1	-	1.99	-0.99	4	0	4	0.000005	0.000001
H300020542	AK5	+	2.18	1.13	3	0	5	0.000017	0.000003
H200020420	MRAS	+	1.87	0.90	4	0	6	0.000031	0.000005
H200000198	PDE3A	-	2.23	-1.15	3	0	7	0.000069	0.00001
H200001173	LARP6	+	1.83	0.87	4	0	8	0.0001	0.000013
H200020677	AK057447	-	1.84	-0.88	4	0	9	0.000334	0.000037
H200015142	NPHP1	+	1.76	0.81	4	0	10	0.00095	0.000095
H300021008	C10orf30	+	1.70	0.77	4	0	11	0.004228	0.000384
H200000466	HLA-DOB	+	1.69	0.76	4	0.000001	12	0.00569	0.000474
H200014087	FAM46C	-	1.73	-0.79	4	0.000001	13	0.009154	0.000704
H300009157	C11orf65	-	1.64	-0.71	3	0.000001	14	0.01001	0.000715
H200011390	LTBP2	+	1.88	0.91	3	0.000001	15	0.010013	0.000668
H200003750	AL109792	-	1.72	-0.78	4	0.000001	16	0.010816	0.000676
H200010238	THBS1	+	1.66	0.73	4	0.000001	17	0.014111	0.00083
H300020839	DUSP19	-	1.71	-0.77	4	0.000001	18	0.014258	0.000792
H200015513	SERPINA3	+	1.84	0.88	3	0.000002	19	0.020623	0.001085
H200008208	ENPP2	-	1.68	-0.75	4	0.000003	20	0.028403	0.00142
H200005825	PRB3	-	1.67	-0.74	4	0.000004	21	0.040675	0.001937
H200021073	TM4SF1	-	1.67	-0.74	4	0.000004	22	0.045841	0.002084
H200002985	SDK1	-	1.86	-0.89	3	0.000006	23	0.062834	0.002732
H200009720	GDF15	+	1.56	0.64	3	0.000008	24	0.082259	0.003427
H200011164	LOX	-	1.80	-0.85	3	0.000009	25	0.092057	0.003682
H200012048	MGC13017	-	1.85	-0.89	3	0.000009	26	0.09624	0.003702
H200017741	TMEM45B	+	1.59	0.67	4	0.000009	27	0.09692	0.00359
H200015613	HSPB9	+	1.73	0.79	3	0.000011	28	0.11411	0.004075

H200010467	AGR2	+	1.58	0.66	4	0.000011	29	0.117516	0.004052
H200020777	C6orf195	-	1.63	-0.70	4	0.000012	30	0.132452	0.004415
H200010727	IRS1	+	1.74	0.80	3	0.000014	31	0.149905	0.004836
H200020020	KCNG1	+	1.74	0.80	3	0.000016	32	0.17553	0.005485
H200002037	C10orf84	-	1.60	-0.68	4	0.000023	33	0.245073	0.007426
H200003683	FABP7	-	1.53	-0.62	3	0.000024	34	0.257166	0.007564
H200000014	PTAFR	+	1.55	0.64	4	0.000025	35	0.267784	0.007651
H200005276	C10orf87	-	1.53	-0.61	3	0.000028	36	0.296434	0.008234
H200009476	PIGZ	+	1.55	0.63	4	0.000031	37	0.324891	0.008781
H200013809	WNT5A	+	1.54	0.62	4	0.000035	38	0.368	0.009684
H300002838	FLJ40288	-	1.76	-0.82	3	0.000037	39	0.394649	0.010119
H200011198	FILIP1	-	1.76	-0.81	3	0.000038	40	0.406603	0.010165
H200001123	ANTXR2	-	1.58	-0.66	4	0.000042	41	0.445791	0.010873
H200008603	SLC15A2	-	1.51	-0.60	3	0.000043	42	0.4528	0.010781
H200000166	IL12A	-	1.57	-0.65	4	0.000049	43	0.522657	0.012155
H200002716	AK5	-	1.50	-0.59	3	0.000056	44	0.601373	0.013668
H200006918	FLJ20152	+	1.52	0.60	4	0.00006	45	0.636429	0.014143
H200011294	MGC12981	+	1.52	0.60	4	0.00006	46	0.643158	0.013982
H200006386	HPGD	-	1.56	-0.65	4	0.00006	47	0.643294	0.013687
H200000469	MPO	-	1.50	-0.59	3	0.000061	48	0.653684	0.013618
H200011026	KRT10	+	1.64	0.71	3	0.000073	49	0.777427	0.015866
H200016574	FRMD1	-	1.69	-0.76	3	0.000073	50	0.779238	0.015585
H200007382	FLJ13224	+	1.64	0.71	3	0.000076	51	0.813262	0.015946
H200011917	COX7A1	-	1.69	-0.76	3	0.000079	52	0.836071	0.016078
H200021283	NANOS1	+	1.51	0.59	4	0.000084	53	0.898714	0.016957
H200014395	BX648202	+	1.51	0.59	4	0.000086	54	0.912232	0.016893
H300022481	OSCAR	-	1.49	-0.57	3	0.000087	55	0.926213	0.01684
H200020889	CXorf43	+	1.50	0.58	4	0.000107	56	1.137821	0.020318
H200003403	FLJ31438	-	1.54	-0.62	4	0.000108	57	1.152097	0.020212
H200012042	LOC91431	-	1.54	-0.62	4	0.00011	58	1.167091	0.020122
H200013014	SLC16A14	+	1.50	0.58	4	0.000115	59	1.219972	0.020677
H300003852	PRKCG	-	1.67	-0.74	3	0.000115	60	1.221696	0.020362
H200019987	PRODH	-	1.66	-0.73	3	0.000124	61	1.317076	0.021591
H200001305	DNAJB9	+	1.49	0.57	4	0.000136	62	1.444517	0.023299

H300018236	TCP11L2	+	1.49	0.57	4	0.000136	63	1.450575	0.023025
H200013802	LHX4	+	1.48	0.57	4	0.000153	64	1.632325	0.025505
H200021151	RBM9	-	1.53	-0.61	4	0.000154	65	1.635209	0.025157
H300002444	C8ORFK36	-	1.52	-0.60	4	0.000178	66	1.895698	0.028723
H200011941	ANGPT2	-	1.64	-0.71	3	0.000194	67	2.064439	0.030813
H200013991	SERAC1	+	1.47	0.56	4	0.000202	68	2.152129	0.031649
H300018985	CEP110	-	1.51	-0.60	4	0.000209	69	2.226466	0.032268
H300011329	MLKL	+	1.47	0.56	4	0.000211	70	2.241321	0.032019
H200010277	LST1	+	1.47	0.56	4	0.00022	71	2.337563	0.032923
H200003227	CMKOR1	+	1.47	0.55	4	0.000231	72	2.458458	0.034145
H200007935	IFT57	-	1.63	-0.70	3	0.000236	73	2.517355	0.034484
H200001545	RUFY3	-	1.51	-0.59	4	0.000257	74	2.737961	0.036999
H200007644	ADAM7	-	1.51	-0.59	4	0.000259	75	2.752208	0.036696
H200006940	CAPG	+	1.46	0.55	4	0.000276	76	2.938156	0.03866
H200017136	CALCB	-	1.50	-0.59	4	0.000284	77	3.02286	0.039258
H200015397	KIAA0980	+	1.45	0.54	4	0.000355	78	3.776347	0.048415
H200001479	C6orf85	+	1.45	0.53	4	0.000382	79	4.068912	0.051505
H200000190	FPR1	-	1.49	-0.57	4	0.000385	80	4.097078	0.051213
H200000879	REG1B	-	1.49	-0.57	4	0.000399	81	4.249685	0.052465
H200015724	TNFSF15	+	1.44	0.53	4	0.000449	82	4.78342	0.058334
H200007868	MYBPC1	-	1.59	-0.67	3	0.000469	83	4.993214	0.060159
H200012140	KCNG1	+	1.44	0.52	4	0.000492	84	5.235449	0.062327
H200005809	GLI3	-	1.62	-0.69	3	0.000494	85	5.261836	0.061904
H200015185	ISL2	+	1.54	0.62	3	0.000495	86	5.272635	0.06131
H200000161	CD40LG	-	1.59	-0.67	3	0.000504	87	5.369869	0.061723
H300000873	MGC43122	-	1.59	-0.67	3	0.000505	88	5.376033	0.061091
H300006130	PLEKHK1	-	1.48	-0.56	4	0.000524	89	5.574902	0.062639
H200013772	HIST1H2BO	-	1.48	-0.56	4	0.00053	90	5.646098	0.062734
H200000656	FMO4	+	1.43	0.52	4	0.00054	91	5.752005	0.063209
H200009530	FLJ31818	+	1.43	0.52	4	0.000574	92	6.10772	0.066388
H200021064	DTNA	+	1.43	0.51	4	0.000631	93	6.713427	0.072187
H200002852	SEC24D	+	1.43	0.51	4	0.000634	94	6.751481	0.071824
H200003464	RAB39B	+	1.42	0.51	4	0.00065	95	6.918969	0.072831
H200003429	ARRDC3	+	1.42	0.51	4	0.000667	96	7.10506	0.074011

H300020762	PEAR1	-	1.57	-0.65	3	0.000669	97	7.119917	0.073401
H300002911	TMEM17	+	1.52	0.61	3	0.000692	98	7.368603	0.07519
H200000084	SPP1	-	1.46	-0.55	4	0.000721	99	7.671138	0.077486
H200011118	C6orf32	-	1.57	-0.65	3	0.00073	100	7.775183	0.077752
H200002219	PLAC1	+	1.40	0.48	3	0.000735	101	7.822932	0.077455
H200000053	AGER	-	1.46	-0.54	4	0.000817	102	8.694815	0.085243
H200015535	HYPE	+	1.42	0.50	4	0.000818	103	8.712005	0.084583
H200017238	GPR135	-	1.46	-0.54	4	0.000833	104	8.872404	0.085312
H200012969	TAF6L	-	1.46	-0.54	4	0.000837	105	8.912978	0.084886
H200006902	KLF10	+	1.41	0.49	4	0.000961	106	10.235835	0.096564
H200005883	GRPR	-	1.45	-0.53	4	0.000986	107	10.498664	0.098118

* (+) indicates upregulation in treated vs untreated and (-) indicates downregulation in treated vs untreated.

† Absolute fold change between untreated and treated cells

‡ Average M of biological replicates

** n = number of biological replicates

†† P = probability of obtaining the observed average M (Z-test)

‡‡ Rank = ranked genes based on obtained probabilities (i.e., the reporter with lowest P will have rank 1)

*** Expected = expected number of reporters calculated as probability times total number of reporters

‡‡‡ FDR = number of expected number of reporters divided by observed number of reporters (rank)
