# Supplementary material for: Tasquinimod (ABR-215050), a quinoline-3-carboxamide anti-angiogenic agent, modulates the expression of thrombospondin-1 in human prostate tumors
Source: Mol Cancer. 2010 May 17;9:107. doi: 10.1186/1476-4598-9-107 (PMC2885345; doi:10.1186/1476-4598-9-107)
Supplement: Additional file 2 — Table S2 - Genes up- or down-regulated at FDR < 0.1 (10%) after in vivo exposure to Tasquinimod for 24 h at 10 mg/kg. The indicated column headings (bold) are explained as follows (also indicated as foot notes): (+) indicates up-regulation in treated vs untreated and (-) indicates down-regulation in treated versus untreated*, Absolute fold change between untreated and treated cells†, Average M of biological replicates‡, n = number of biological replicates**, P = probability of obtaining the observed average M (Z-test) ††, Rank = ranked genes based on obtained probabilities (i.e., the reporter with lowest P will have rank 1) ‡‡, Expected = expected number of reporters calculated as probability times total number of reporters ***, and FDR = number of expected number of reporters divided by observed number of reporters (rank) ‡‡‡. [file 1476-4598-9-107-S2.PDF]

Additional File 2, Table S2.

Genes up- or down-regulated at FDR< 0.1 (10%) after 24h in vivo exposure to Tasquinimod at 10 mg/kg.

reporterId	geneSymbol	up/down*	Fold change†	Average M‡	n**	P††	Rank‡‡	Expected***	FDR‡‡‡
H200000106	ADM	-	3.78	-1.92	4	0	1	0	0
H300003327	CYP1A2	+	39.74	5.31	4	0	2	0	0
H300006484	CYP1A1	+	45.94	5.52	4	0	3	0	0
H300012264	CA3	+	6.76	2.76	4	0	4	0	0
H300002326	HLA-DMB	+	3.10	1.63	4	0	5	0	0
H300017501	CD74	+	3.97	1.99	4	0	6	0	0
H300012307	VEGF	-	2.93	-1.55	4	0	7	0	0
H300002875	TRIOBP	-	2.79	-1.48	4	0	8	0	0
H300011320	STC2	-	2.72	-1.45	4	0	9	0	0
H300016030	FOXN4	+	2.65	1.40	4	0	10	0	0
H200006197	NDRG1	-	2.65	-1.40	4	0	11	0	0
H300018901	SLC11A1	+	2.60	1.38	4	0	12	0	0
H200015120	MYLK	+	2.58	1.37	4	0	13	0	0
H200010317	CXCR4	-	2.58	-1.37	4	0	14	0	0
H200000068	XDH	+	2.55	1.35	4	0	15	0	0
H300019354	REG4	+	2.50	1.32	4	0	16	0.000001	0
H200006783	UCP2	+	2.46	1.30	4	0	17	0.000002	0
H300014518	ERO1L	-	2.46	-1.30	4	0	18	0.000003	0
H200014799	PFKFB4	-	2.45	-1.29	4	0	19	0.000003	0
H300021283	PFKFB3	-	2.44	-1.29	4	0	20	0.000004	0
H300021585	C21orf29	+	2.42	1.28	4	0	21	0.000004	0
H300022120	FOXN4	+	2.41	1.27	4	0	22	0.000005	0
H300016091	PLOD2	-	2.37	-1.25	4	0	23	0.000014	0.000001
H300019070	LIPE	+	2.35	1.23	4	0	24	0.000018	0.000001
H200008321	FLJ22662	+	2.71	1.44	3	0	25	0.000023	0.000001
H300018429	ERO1L	-	2.33	-1.22	4	0	26	0.000034	0.000001
H200007994	BHLHB2	-	2.30	-1.20	4	0	27	0.000061	0.000002
H200001663	NUPR1	-	2.28	-1.19	4	0	28	0.000086	0.000003

H300021765	VEGF	-	2.27	-1.18	4	0	29	0.000107	0.000004
H300002696	ABTB2	-	2.24	-1.16	4	0	30	0.000183	0.000006
H300020472	FRMD4A	+	2.22	1.15	4	0	31	0.000229	0.000007
H200004751	ERG	+	2.20	1.14	4	0	32	0.000294	0.000009
H200015078	SCIN	+	2.18	1.13	4	0	33	0.00044	0.000013
H200010069	EOMES	-	2.19	-1.13	4	0	34	0.000475	0.000014
H200016081	HTR5A	-	2.19	-1.13	4	0	35	0.000477	0.000014
H300013567	SSPN	-	2.18	-1.13	4	0	36	0.000525	0.000015
H300019320	SULF2	+	2.16	1.11	4	0	37	0.000618	0.000017
H300020690	ARHGAP30	+	2.15	1.10	4	0	38	0.000827	0.000022
H200009250	LSM16	+	2.15	1.10	4	0	39	0.000852	0.000022
H200000024	IL2RG	+	2.15	1.10	4	0	40	0.000859	0.000021
H200004515	PLOD2	-	2.15	-1.11	4	0	41	0.000882	0.000022
H300007366	SCNN1G	-	2.15	-1.11	4	0	42	0.000912	0.000022
H200005436	CXorf9	+	2.14	1.10	4	0	43	0.000955	0.000022
H200013227	PLD4	+	2.14	1.10	4	0	44	0.001037	0.000024
H300021766	VEGF	-	2.14	-1.10	4	0	45	0.001139	0.000025
H300022481	OSCAR	-	2.13	-1.09	4	0	46	0.001406	0.000031
H200005452	HIG2	-	2.12	-1.08	4	0	47	0.0017	0.000036
H300019247	LRCH2	-	2.19	-1.13	3	0	48	0.001709	0.000036
H300022039	JMJD1A	-	2.12	-1.08	4	0	49	0.001781	0.000036
H300013127	FOXN4	+	2.20	1.14	3	0	50	0.001847	0.000037
H300012065	ABTB2	-	2.11	-1.08	4	0	51	0.001916	0.000038
H300007360	IGFBP3	-	2.10	-1.07	4	0	52	0.00239	0.000046
H200015902	SSPO	-	2.10	-1.07	4	0	53	0.002399	0.000045
H200016114	KRTHA6	+	2.08	1.05	4	0	54	0.003243	0.00006
H200017994	STAB1	+	2.07	1.05	4	0	55	0.003504	0.000064
H300001168	GPR153	-	2.08	-1.06	4	0	56	0.003584	0.000064
H300006745	FGD2	+	2.05	1.04	4	0	57	0.004972	0.000087
H300014139	LGP2	+	2.03	1.02	4	0	58	0.007301	0.000126
H200006940	CAPG	+	2.03	1.02	4	0	59	0.008159	0.000138
H300008121	PIWIL4	+	2.02	1.01	4	0.000001	60	0.009743	0.000162
H300019395	CD36	+	2.02	1.01	4	0.000001	61	0.009947	0.000163
H300011129	RUTBC2	-	2.02	-1.02	4	0.000001	62	0.010011	0.000161

H200003548	FOS	-	2.02	-1.02	4	0.000001	63	0.010217	0.000162
H200011164	LOX	-	2.01	-1.01	4	0.000001	64	0.012233	0.000191
H200005388	HMGCS2	+	2.00	1.00	4	0.000001	65	0.012581	0.000194
H200017605	ZIC5	+	2.00	1.00	4	0.000001	66	0.013998	0.000212
H200006322	P4HA1	-	2.00	-1.00	4	0.000001	67	0.014431	0.000215
H200010086	EGLN1	-	2.00	-1.00	4	0.000001	68	0.01594	0.000234
H200000676	JUND	-	1.99	-0.99	4	0.000001	69	0.017504	0.000254
H300002229	ENG	+	1.98	0.98	4	0.000001	70	0.019613	0.00028
H300021037	ABTB2	-	1.99	-0.99	4	0.000001	71	0.019914	0.00028
H200003830	PPP1R3B	-	1.99	-0.99	4	0.000001	72	0.020028	0.000278
H300011291	VEGF	-	1.98	-0.99	4	0.000001	73	0.020181	0.000276
H300013064	PRKCB1	+	1.98	0.98	4	0.000001	74	0.020602	0.000278
H300008679	EGLN3	-	1.97	-0.98	4	0.000001	75	0.024958	0.000333
H200008441	URP2	+	1.96	0.97	4	0.000001	76	0.026278	0.000346
H300021589	FMNL1	+	1.95	0.96	4	0.000002	77	0.032404	0.000421
H300022055	NRP2	+	1.95	0.96	4	0.000002	78	0.032452	0.000416
H300020465	VAV3	+	1.94	0.96	4	0.000002	79	0.03796	0.000481
H300013084	MYH9	-	1.95	-0.96	4	0.000002	80	0.039833	0.000498
H300022053	FAM13A1	-	1.94	-0.96	4	0.000002	81	0.042453	0.000524
H200014052	ALDOC	-	1.94	-0.95	4	0.000003	82	0.04914	0.000599
H200013895	PPFIA4	-	1.94	-0.95	4	0.000003	83	0.049535	0.000597
H200004267	CERK	+	1.93	0.95	4	0.000003	84	0.050345	0.000599
H300013598	DUSP4	-	2.19	-1.13	3	0.000003	85	0.053155	0.000625
H300019826	P4HA1	-	1.93	-0.95	4	0.000003	86	0.056514	0.000657
H300021049	PARVA	+	1.92	0.94	4	0.000003	87	0.05656	0.00065
H300006468	PDK1	-	1.93	-0.95	4	0.000003	88	0.057836	0.000657
H300016095	NRP2	+	1.92	0.94	4	0.000003	89	0.060935	0.000685
H300019416	EGLN1	-	1.92	-0.94	4	0.000003	90	0.066921	0.000744
H300018073	INSIG2	-	1.91	-0.93	4	0.000004	91	0.080832	0.000888
H300019814	CD44	+	1.90	0.92	4	0.000004	92	0.085161	0.000926
H300013591	ARHGAP30	+	1.97	0.98	3	0.000004	93	0.086004	0.000925
H300021461	EMR1	+	1.89	0.92	4	0.000005	94	0.09514	0.001012
H300019007	SMAD9	-	1.90	-0.92	4	0.000005	95	0.096174	0.001012
H300000314	ENO2	-	1.90	-0.92	4	0.000005	96	0.096589	0.001006

H300021959	PARP6	+	1.89	0.92	4	0.000005	97	0.09954	0.001026
H300020149	CDH13	+	1.89	0.92	4	0.000005	98	0.099683	0.001017
H200001820	GADD45G	-	1.89	-0.92	4	0.000006	99	0.105915	0.00107
H300015164	PSMA8	-	1.89	-0.92	4	0.000006	100	0.109196	0.001092
H200001656	RPP25	+	1.87	0.90	4	0.000008	101	0.146353	0.001449
H200014390	WSB1	-	1.87	-0.91	4	0.000008	102	0.147565	0.001447
H300022903	SCAND2	-	1.87	-0.90	4	0.000008	103	0.161785	0.001571
H300000366	FLJ00292	+	1.86	0.89	4	0.000009	104	0.177615	0.001708
H300010370	VNN1	+	1.93	0.95	3	0.000009	105	0.177785	0.001693
H300022773	SCAND2	-	1.86	-0.89	4	0.00001	106	0.192616	0.001817
H300018793	PAX2	+	1.85	0.89	4	0.000011	107	0.20318	0.001899
H300012209	VLDLR	-	1.86	-0.89	4	0.000011	108	0.208515	0.001931
H300003672	ENO2	-	1.85	-0.89	4	0.000011	109	0.218625	0.002006
H300017196	LMBR1L	-	1.84	-0.88	4	0.000013	110	0.253375	0.002303
H200001658	RAPGEF3	+	1.83	0.87	4	0.000014	111	0.266761	0.002403
H300010421	SCAND2	-	1.84	-0.88	4	0.000014	112	0.270551	0.002416
H200001807	C1QA	+	1.83	0.87	4	0.000015	113	0.282156	0.002497
H200011421	CDKN1C	-	1.84	-0.88	4	0.000015	114	0.283241	0.002485
H200020286	BC014381	-	1.84	-0.88	4	0.000015	115	0.290069	0.002522
H200007178	MYLK2	+	1.83	0.87	4	0.000016	116	0.301116	0.002596
H200000236	LTB	+	1.83	0.87	4	0.000016	117	0.306951	0.002624
H200004411	KIAA0922	+	1.83	0.87	4	0.000016	118	0.308506	0.002614
H200010483	GABRA2	-	1.83	-0.87	4	0.000016	119	0.310931	0.002613
H200008584	ASB2	-	2.02	-1.01	3	0.000017	120	0.323169	0.002693
H200011342	ENC1	-	1.83	-0.87	4	0.000017	121	0.32398	0.002678
H200015399	LGALS1	+	1.82	0.87	4	0.000017	122	0.326282	0.002674
H300018421	SEMA3B	-	1.83	-0.87	4	0.000018	123	0.352435	0.002865
H300005146	EDARADD	-	1.82	-0.87	4	0.000019	124	0.356582	0.002876
H300001588	ASGR1	-	1.82	-0.86	4	0.00002	125	0.379308	0.003034
H300007039	ARL10	+	1.81	0.86	4	0.000021	126	0.404173	0.003208
H300020247	PPFIA4	-	1.82	-0.86	4	0.000022	127	0.415575	0.003272
H300017880	PLOD2	-	1.81	-0.86	4	0.000022	128	0.426225	0.00333
H300017441	RBMS1	+	1.81	0.85	4	0.000022	129	0.427312	0.003312
H300012695	TRAPPC4	+	1.81	0.85	4	0.000023	130	0.434753	0.003344

H300017770	TBC1D9B	-	1.81	-0.86	4	0.000024	131	0.451612	0.003447
H200011082	PPARG	+	1.80	0.85	4	0.000024	132	0.461358	0.003495
H200017637	PDE7B	+	1.80	0.84	4	0.000027	133	0.521709	0.003923
H300007547	TSHZ2	+	1.79	0.84	4	0.000028	134	0.53836	0.004018
H300004389	JMJD3	-	1.80	-0.85	4	0.000028	135	0.54372	0.004028
H200003765	ZNF395	-	1.80	-0.85	4	0.000029	136	0.547186	0.004023
H300018425	ACPP	-	1.80	-0.85	4	0.000029	137	0.557887	0.004072
H200001605	EPAS1	+	1.79	0.84	4	0.000032	138	0.614065	0.00445
H300002444	C8ORFK36	-	1.79	-0.84	4	0.000032	139	0.614608	0.004422
H200005085	PPYR1	-	1.79	-0.84	4	0.000032	140	0.620849	0.004435
H300010663	SLC15A3	+	1.99	0.99	3	0.000033	141	0.625265	0.004435
H200004354	ATP4A	-	1.79	-0.84	4	0.000034	142	0.643166	0.004529
H200013157	IGSF6	-	1.79	-0.84	4	0.000034	143	0.651785	0.004558
H200017080	HSPA1B	-	1.79	-0.84	4	0.000035	144	0.662168	0.004598
H200019847	DDIT3	-	1.79	-0.84	4	0.000035	145	0.675269	0.004657
H300016697	GSDMDC1	+	1.78	0.83	4	0.000036	146	0.686378	0.004701
H200020363	SPTBN1	+	1.78	0.83	4	0.000039	147	0.744495	0.005065
H200006193	NR3C1	-	1.78	-0.83	4	0.000039	148	0.752998	0.005088
H200002187	TIPARP	+	1.77	0.83	4	0.00004	149	0.77542	0.005204
H200020407	MST1	-	1.78	-0.83	4	0.000041	150	0.785164	0.005234
H300001396	THEM5	-	1.78	-0.83	4	0.000042	151	0.797059	0.005279
H300008083	PTCH1	+	1.77	0.82	4	0.000042	152	0.813405	0.005351
H200010547	CDH10	-	2.01	-1.01	3	0.000043	153	0.829175	0.005419
H200010916	KSP37	+	1.96	0.97	3	0.000044	154	0.834984	0.005422
H300016264	ZBTB10	-	1.77	-0.83	4	0.000045	155	0.866044	0.005587
H200003541	STC1	-	1.77	-0.83	4	0.000046	156	0.878943	0.005634
H200009840	VIM	+	1.76	0.82	4	0.000048	157	0.917333	0.005843
H200010154	HES6	-	1.77	-0.82	4	0.000048	158	0.925036	0.005855
H200016029	HOXC8	+	1.95	0.96	3	0.000048	159	0.925672	0.005822
H200012008	SCGN	+	1.76	0.81	4	0.000051	160	0.983836	0.006149
H300002310	RP11-393I2.3	-	1.77	-0.82	4	0.000052	161	1.000687	0.006215
H200016770	MFI2	-	1.76	-0.82	4	0.000053	162	1.010793	0.006239
H300015677	ZNF511	-	1.76	-0.82	4	0.000053	163	1.014502	0.006224
H300000058	FUT11	-	1.76	-0.82	4	0.000055	164	1.060415	0.006466

H300011209	IGSF4B	+	1.75	0.81	4	0.000057	165	1.103222	0.006686
H300016144	PKM2	-	1.96	-0.97	3	0.000059	166	1.1277	0.006793
H300008608	CPA1	-	1.80	-0.85	3	0.000059	167	1.13868	0.006818
H200012191	IGFBP7	+	1.75	0.81	4	0.000059	168	1.140508	0.006789
H200001687	CHST2	-	1.76	-0.81	4	0.00006	169	1.148086	0.006793
H300010544	HLA-DQB2	-	1.80	-0.84	3	0.000063	170	1.215514	0.00715
H300020454	HNMT	+	1.75	0.80	4	0.000065	171	1.253831	0.007332
H300005207	UCN3	+	1.74	0.80	4	0.000066	172	1.274149	0.007408
H300020404	PAPSS2	+	1.74	0.80	4	0.000069	173	1.315671	0.007605
H300012877	ARHGAP24	+	1.74	0.80	4	0.00007	174	1.348528	0.00775
H200018156	APLN	-	1.75	-0.80	4	0.000073	175	1.397	0.007983
H200010623	FOXJ1	-	1.75	-0.80	4	0.000074	176	1.410562	0.008015
H200012285	BRDG1	+	1.74	0.80	4	0.000074	177	1.411224	0.007973
H300003255	DKFZp686B0	-	1.75	-0.80	4	0.000074	178	1.421863	0.007988
H300019853	ABCB11	+	1.74	0.80	4	0.000077	179	1.478557	0.00826
H200006772	DPT	+	1.73	0.79	4	0.00008	180	1.542088	0.008567
H300021469	AMZ1	+	1.73	0.79	4	0.000084	181	1.609172	0.00889
H300020167	SLC14A1	+	1.73	0.79	4	0.000087	182	1.668293	0.009166
H200004548	KRT32	+	1.73	0.79	4	0.000087	183	1.676236	0.00916
H300021354	FLJ00264	-	1.73	-0.79	4	0.000088	184	1.695547	0.009215
H200013379	KIAA1602	+	1.73	0.79	4	0.000089	185	1.714593	0.009268
H300006144	DOCK2	+	1.73	0.79	4	0.000089	186	1.716489	0.009228
H300017296	P4HA1	-	1.73	-0.79	4	0.000091	187	1.747543	0.009345
H200003669	MGC17330	-	1.73	-0.79	4	0.000092	188	1.771024	0.00942
H300014044	CTSC	+	1.72	0.79	4	0.000093	189	1.783073	0.009434
H200000546	WAS	+	1.72	0.78	4	0.000102	190	1.947515	0.01025
H300013743	ELMO1	+	1.72	0.78	4	0.000105	191	2.019594	0.010574
H200016284	NMUR1	-	1.72	-0.79	4	0.000105	192	2.023775	0.01054
H300007284	OSGIN1	+	1.72	0.78	4	0.000105	193	2.023953	0.010487
H200006105	SPARCL1	+	1.72	0.78	4	0.000106	194	2.038904	0.01051
H200001357	INSIG2	-	1.72	-0.78	4	0.000108	195	2.075236	0.010642
H200000807	P4HA2	-	1.72	-0.78	4	0.000111	196	2.132888	0.010882
H200012631	SRPX2	+	1.89	0.92	3	0.000111	197	2.139207	0.010859
H300020872	ANKZF1	-	1.72	-0.78	4	0.000118	198	2.273441	0.011482

H200007072	AMPD3	-	1.72	-0.78	4	0.000123	199	2.360421	0.011861
H300021273	NAALAD2	+	1.89	0.92	3	0.000124	200	2.372585	0.011863
H300019525	NAB1	+	1.71	0.77	4	0.000127	201	2.433147	0.012105
H200013710	C12orf24	-	1.71	-0.77	4	0.000136	202	2.604157	0.012892
H200004736	DPP4	+	1.70	0.77	4	0.000136	203	2.604184	0.012828
H300013614	PIAS2	-	1.71	-0.77	4	0.000136	204	2.615585	0.012821
H300014915	STC2	-	1.71	-0.77	4	0.000141	205	2.705319	0.013197
H300002281	PTCH1	+	1.70	0.77	4	0.000143	206	2.740554	0.013304
H200017356	PIK3R5	+	1.70	0.76	4	0.000144	207	2.767053	0.013367
H300006121	DST	+	1.70	0.76	4	0.000148	208	2.846922	0.013687
H200000225	IL8RB	-	1.70	-0.77	4	0.000151	209	2.887931	0.013818
H300018715	PLAT	+	1.70	0.76	4	0.000152	210	2.907609	0.013846
H200002626	DHRS3	-	1.70	-0.77	4	0.000153	211	2.94163	0.013941
H300012086	SLC38A3	-	1.70	-0.77	4	0.000155	212	2.971067	0.014014
H300001823	NMB	-	1.70	-0.77	4	0.000158	213	3.038138	0.014264
H300020689	SLC26A9	+	1.75	0.81	3	0.00016	214	3.074354	0.014366
H300001770	C8orf31	+	1.69	0.76	4	0.000162	215	3.109468	0.014463
H200012131	MXI1	-	1.70	-0.76	4	0.000165	216	3.157059	0.014616
H200001766	OLFML3	+	1.69	0.76	4	0.000167	217	3.202849	0.01476
H200012140	KCNG1	+	1.69	0.76	4	0.000167	218	3.210125	0.014725
H200003373	WIPF1	+	1.68	0.75	4	0.000183	219	3.514649	0.016049
H200000102	FLT3	+	1.68	0.75	4	0.000184	220	3.529058	0.016041
H300017278	HYAL2	-	1.87	-0.90	3	0.000194	221	3.715882	0.016814
H200014523	PPP1R14C	-	1.72	-0.78	3	0.000201	222	3.863582	0.017404
H300016158	WDR66	-	1.68	-0.75	4	0.00021	223	4.031638	0.018079
H200012443	SMAD9	-	1.68	-0.75	4	0.000211	224	4.053091	0.018094
H300012416	KCNAB2	-	1.68	-0.75	4	0.000216	225	4.152627	0.018456
H300011466	BTK	+	1.67	0.74	4	0.000226	226	4.338956	0.019199
H300018592	ANGPT2	+	1.67	0.74	4	0.000227	227	4.359694	0.019206
H300012218	PTHR2	-	1.72	-0.78	3	0.000227	228	4.36432	0.019142
H300019004	HOXB2	+	1.67	0.74	4	0.000236	229	4.534756	0.019802
H200006288	ALDH1A1	+	1.67	0.74	4	0.000238	230	4.564789	0.019847
H300014920	KLHL24	-	1.67	-0.74	4	0.000243	231	4.656566	0.020158
H200011334	TMEM45A	-	1.67	-0.74	4	0.000249	232	4.778949	0.020599

H200011718	TNRC9	+	1.66	0.74	4	0.000256	233	4.919456	0.021114
H300000628	DMRTA1	+	1.66	0.74	4	0.000256	234	4.921164	0.021031
H300002206	ST8SIA3	-	1.87	-0.90	3	0.000259	235	4.967263	0.021137
H300011648	PHLDB2	+	1.66	0.73	4	0.000262	236	5.032329	0.021323
H300010492	MLL5	-	1.67	-0.74	4	0.000265	237	5.077211	0.021423
H200011059	ALOX5AP	+	1.66	0.73	4	0.000268	238	5.132988	0.021567
H200011761	ARL4C	+	1.66	0.73	4	0.000268	239	5.14368	0.021522
H200009501	GSN	+	1.66	0.73	4	0.00027	240	5.174466	0.02156
H200002762	DHRS10	-	1.67	-0.74	4	0.000272	241	5.218196	0.021652
H300013374	PRRX1	+	1.66	0.73	4	0.000273	242	5.230228	0.021613
H300014377	KIFC3	+	1.66	0.73	4	0.000276	243	5.292245	0.021779
H300015679	FUT8	-	1.67	-0.74	4	0.000278	244	5.342388	0.021895
H300020281	VGLL4	-	1.67	-0.74	4	0.000281	245	5.399881	0.02204
H200001083	CBX4	-	1.67	-0.74	4	0.000282	246	5.407439	0.021981
H300019276	PDLIM4	+	1.66	0.73	4	0.000284	247	5.453301	0.022078
H200013473	EFNB1	-	1.66	-0.73	4	0.000291	248	5.579706	0.022499
H300019277	MGAT1	-	1.66	-0.73	4	0.000295	249	5.663551	0.022745
H300021484	WDR66	-	1.66	-0.73	4	0.000304	250	5.841016	0.023364
H200017363	IL17C	+	1.65	0.73	4	0.000306	251	5.866105	0.023371
H200000453	INHA	+	1.65	0.72	4	0.000316	252	6.062193	0.024056
H300005893	CRIP3	+	1.65	0.72	4	0.000336	253	6.448974	0.02549
H200007876	SLC2A1	-	1.65	-0.73	4	0.000339	254	6.511539	0.025636
H300019391	TAOK3	+	1.65	0.72	4	0.00034	255	6.526543	0.025594
H300020857	ANTXR2	+	1.65	0.72	4	0.000355	256	6.807635	0.026592
H300006978	HIST1H4D	-	1.65	-0.72	4	0.000381	257	7.308441	0.028438
H200006338	PDIA5	+	1.79	0.84	3	0.000385	258	7.387014	0.028632
H300014720	SLC6A6	-	1.64	-0.72	4	0.000399	259	7.665049	0.029595
H300001940	AP3B1	+	1.64	0.71	4	0.0004	260	7.670201	0.029501
H300015458	SYK	+	1.64	0.71	4	0.000408	261	7.832697	0.03001
H300020516	BANK1	+	1.64	0.71	4	0.000415	262	7.954665	0.030361
H300008222	TFDP3	-	1.81	-0.85	3	0.000415	263	7.968317	0.030298
H300003608	FUT5	-	1.83	-0.87	3	0.000418	264	8.021058	0.030383
H200019656	LOXL3	+	1.64	0.71	4	0.00042	265	8.058963	0.030411
H300010817	FAP	+	1.63	0.71	4	0.000421	266	8.084372	0.030392

H200007984	DUSP1	-	1.78	-0.83	3	0.000426	267	8.164889	0.03058
H200004397	COPZ2	+	1.63	0.71	4	0.000428	268	8.210726	0.030637
H300020238	EFNA3	-	1.64	-0.71	4	0.000433	269	8.312075	0.0309
H300006205	S100P	+	1.63	0.71	4	0.000435	270	8.342598	0.030899
H200013635	EBF2	-	1.67	-0.74	3	0.000437	271	8.392107	0.030967
H300019933	ITGA9	+	1.63	0.71	4	0.000438	272	8.400241	0.030883
H300010387	MALT1	-	1.80	-0.85	3	0.00044	273	8.451838	0.030959
H300006743	ERN2	+	1.63	0.71	4	0.000441	274	8.469248	0.03091
H300020917	GUCY1A3	+	1.63	0.71	4	0.000448	275	8.595691	0.031257
H300000863	MYOD1	-	1.64	-0.71	4	0.000451	276	8.653847	0.031355
H300006659	TMEM92	-	1.64	-0.71	4	0.000456	277	8.748305	0.031582
H200007794	ACRV1	+	1.63	0.70	4	0.000457	278	8.770743	0.031549
H300020373	TRIM55	-	1.64	-0.71	4	0.000461	279	8.843917	0.031699
H300002655	AGXT	+	1.63	0.70	4	0.000472	280	9.056454	0.032344
H300012657	LGALS1	+	1.63	0.70	4	0.000483	281	9.266347	0.032976
H300003321	CREB3L1	+	1.63	0.70	4	0.000487	282	9.350662	0.033158
H300020646	MMP14	+	1.62	0.70	4	0.000508	283	9.742096	0.034424
H200014355	FAM111B	+	1.62	0.70	4	0.000513	284	9.849435	0.034681
H200014093	VGLL4	-	1.63	-0.70	4	0.000517	285	9.919217	0.034804
H200006664	NFIL3	-	1.63	-0.70	4	0.000524	286	10.048618	0.035135
H300018985	CEP110	+	1.67	0.74	3	0.000528	287	10.130914	0.035299
H300012774	ELF5	-	1.63	-0.70	4	0.000532	288	10.199215	0.035414
H300011329	MLKL	+	1.62	0.70	4	0.000533	289	10.226614	0.035386
H300011991	LPIN1	-	1.63	-0.70	4	0.000534	290	10.237379	0.035301
H300008317	SMPDL3A	-	1.62	-0.70	4	0.000588	291	11.273705	0.038741
H300004275	CNTNAP2	+	1.61	0.69	4	0.000588	292	11.280968	0.038633
H200002927	CYP39A1	+	1.61	0.69	4	0.000594	293	11.39668	0.038897
H300003509	BC042851	-	1.62	-0.70	4	0.000594	294	11.399908	0.038775
H200000454	INHBB	+	1.61	0.69	4	0.000605	295	11.601304	0.039326
H300006456	HIST1H3B	+	1.61	0.69	4	0.000609	296	11.683828	0.039472
H200006290	PLA2G2A	-	1.62	-0.69	4	0.000613	297	11.765336	0.039614
H300003254	TM4SF1	+	1.61	0.69	4	0.000626	298	12.017429	0.040327
H300022473	MAL	-	1.77	-0.82	3	0.000645	299	12.380456	0.041406
H200005513	CA9	-	1.62	-0.69	4	0.000647	300	12.414476	0.041382

H300013964	GPM6B	+	1.61	0.69	4	0.000654	301	12.547836	0.041687
H300011920	UBC	-	1.61	-0.69	4	0.000657	302	12.605576	0.04174
H300019597	RHOJ	+	1.61	0.68	4	0.000675	303	12.943044	0.042716
H200010038	AADAT	+	1.61	0.68	4	0.000681	304	13.074965	0.04301
H300012876	SMOX	+	1.61	0.68	4	0.000685	305	13.135326	0.043067
H200004533	BATF	-	1.77	-0.82	3	0.000685	306	13.139172	0.042938
H300008949	DUSP5	-	1.61	-0.69	4	0.000688	307	13.198849	0.042993
H200001249	SLC17A7	-	1.61	-0.69	4	0.000691	308	13.250011	0.04302
H300014718	DUSP6	+	1.60	0.68	4	0.000691	309	13.251741	0.042886
H300021172	HOP	+	1.60	0.68	4	0.000692	310	13.275506	0.042824
H300014144	YEATS2	-	1.74	-0.80	3	0.000708	311	13.583115	0.043676
H200007038	SOX4	-	1.61	-0.69	4	0.000714	312	13.707874	0.043935
H200017722	CPA6	+	1.60	0.68	4	0.000715	313	13.710939	0.043805
H200017004	DSCR1L2	+	1.60	0.68	4	0.000715	314	13.711223	0.043666
H300018591	ESR1	+	1.60	0.68	4	0.000715	315	13.721487	0.04356
H300020036	ANK1	+	1.74	0.80	3	0.000722	316	13.855321	0.043846
H300007725	FAM80A	-	1.61	-0.69	4	0.000723	317	13.88033	0.043787
H200005556	CLIC3	-	1.61	-0.68	4	0.000753	318	14.4487	0.045436
H300014004	GRAMD1B	+	1.60	0.68	4	0.000756	319	14.497734	0.045447
H300008871	CHAC2	+	1.60	0.68	4	0.000758	320	14.545941	0.045456
H200016300	PSMA4	+	1.60	0.68	4	0.000762	321	14.625194	0.045561
H300015527	VNN2	+	1.74	0.80	3	0.000765	322	14.685098	0.045606
H300020008	OSBPL3	-	1.60	-0.68	4	0.000772	323	14.808427	0.045847
H300016227	PFKP	-	1.60	-0.68	4	0.000808	324	15.49482	0.047824
H300010900	RUNX2	+	1.59	0.67	4	0.000812	325	15.574404	0.047921
H200015881	ITIH5L	-	1.60	-0.68	4	0.000813	326	15.604126	0.047865
H300019843	TTC7A	-	1.60	-0.68	4	0.000819	327	15.710446	0.048044
H300016845	FLJ22671	+	1.59	0.67	4	0.000822	328	15.774997	0.048095
H300018157	AGPAT1	-	1.63	-0.71	3	0.000822	329	15.776808	0.047954
H300005599	LOC153222	-	1.60	-0.68	4	0.000827	330	15.865789	0.048078
H300006966	SKIP	+	1.59	0.67	4	0.000828	331	15.881557	0.047981
H300016077	WSCD1	+	1.59	0.67	4	0.000857	332	16.441241	0.049522
H200005761	PYCARD	-	1.75	-0.81	3	0.00086	333	16.491317	0.049523
H200006597	MEF2C	+	1.59	0.67	4	0.000862	334	16.540669	0.049523

H300016072	PPP2R5B	-	1.60	-0.68	4	0.000866	335	16.615978	0.0496
H200014582	LRRC8C	+	1.59	0.67	4	0.000878	336	16.845754	0.050136
H300014092	HORMAD1	-	1.60	-0.67	4	0.000878	337	16.84915	0.049997
H300000419	AK095133	-	1.63	-0.70	3	0.000891	338	17.092598	0.05057
H200012198	RASA3	+	1.59	0.67	4	0.000902	339	17.306212	0.051051
H300012090	MGAT1	-	1.59	-0.67	4	0.000915	340	17.55445	0.051631
H300007309	MAN1C1	-	1.59	-0.67	4	0.000916	341	17.581649	0.051559
H300013715	APBB3	-	1.59	-0.67	4	0.000927	342	17.785742	0.052005
H300013713	C9orf84	+	1.59	0.67	4	0.000932	343	17.885397	0.052144
H300019397	C6orf148	-	1.59	-0.67	4	0.000933	344	17.898853	0.052032
H300000343	FAM78B	+	1.59	0.66	4	0.00095	345	18.22129	0.052815
H300020196	ZNF160	-	1.59	-0.67	4	0.000954	346	18.296872	0.052881
H200004042	ARRDC2	-	1.59	-0.67	4	0.000984	347	18.87395	0.054392
H200000529	VDR	+	1.58	0.66	4	0.000987	348	18.945086	0.05444
H200019615	SLN	+	1.58	0.66	4	0.001005	349	19.283284	0.055253
H300012271	ZNF318	+	1.58	0.66	4	0.001006	350	19.29255	0.055122
H200017074	OSBPL7	+	1.58	0.66	4	0.001006	351	19.29595	0.054974
H300008660	MYLPF	+	1.58	0.66	4	0.001009	352	19.350734	0.054974
H300010428	GALNTL1	+	1.58	0.66	4	0.001025	353	19.660319	0.055695
H300022478	HIG2	-	1.59	-0.67	4	0.001038	354	19.914004	0.056254
H300021571	FMNL1	+	1.58	0.66	4	0.001047	355	20.087896	0.056586
H200013042	NCKAP1L	+	1.58	0.66	4	0.001061	356	20.358189	0.057186
H300006529	FBXO39	+	1.58	0.66	4	0.001089	357	20.890382	0.058516
H300009417	C6orf190	-	1.73	-0.79	3	0.001093	358	20.963927	0.058558
H200017760	C20orf10	-	1.73	-0.79	3	0.001099	359	21.091673	0.058751
H300021012	UAP1L1	-	1.58	-0.66	4	0.001133	360	21.740481	0.06039
H200004964	KIAA1199	+	1.57	0.65	4	0.001149	361	22.048721	0.061077
H300019273	SH3BP5	+	1.57	0.65	4	0.001162	362	22.295582	0.06159
H200013889	RPGR	+	1.57	0.65	4	0.001171	363	22.470881	0.061903
H300018416	FYN	+	1.57	0.65	4	0.001195	364	22.934382	0.063007
H300014943	GSN	+	1.57	0.65	4	0.001221	365	23.436792	0.06421
H200017198	S100A6	+	1.57	0.65	4	0.001223	366	23.464403	0.06411
H300018508	ACACB	+	1.57	0.65	4	0.00124	367	23.796988	0.064842
H300002856	DSG1	-	1.57	-0.66	4	0.001243	368	23.852971	0.064818

H300022345	KRTHB6	+	1.57	0.65	4	0.00125	369	23.990292	0.065014
H200004991	GNA14	+	1.61	0.69	3	0.001257	370	24.114289	0.065174
H300007520	ADAMTS15	+	1.70	0.76	3	0.001278	371	24.514541	0.066077
H300021006	PPIL5	+	1.57	0.65	4	0.001279	372	24.531063	0.065944
H200011012	CD70	-	1.57	-0.65	4	0.001312	373	25.173539	0.067489
H200013795	SGCD	+	1.56	0.65	4	0.001314	374	25.214161	0.067418
H200005899	EPHA4	+	1.56	0.64	4	0.001346	375	25.820916	0.068856
H300004006	MRM1	+	1.56	0.64	4	0.001413	376	27.105438	0.072089
H200009536	S100A1	-	1.57	-0.65	4	0.001424	377	27.316673	0.072458
H300015598	SCNN1B	-	1.68	-0.75	3	0.001428	378	27.405303	0.072501
H200012359	RUNX2	+	1.56	0.64	4	0.001455	379	27.913585	0.073651
H300016409	MKNK2	-	1.56	-0.65	4	0.001458	380	27.967788	0.073599
H300009602	GBE1	-	1.56	-0.65	4	0.00146	381	28.017162	0.073536
H300016321	KLHL24	-	1.56	-0.65	4	0.001462	382	28.056643	0.073447
H300015970	TREH	+	1.56	0.64	4	0.001465	383	28.101469	0.073372
H200000600	DUSP4	-	1.56	-0.64	4	0.00149	384	28.597037	0.074471
H300021031	SERPINA1	-	1.56	-0.64	4	0.001499	385	28.763327	0.07471
H300021769	APBB3	-	1.56	-0.64	4	0.0015	386	28.777322	0.074553
H300018890	C3orf28	-	1.56	-0.64	4	0.001515	387	29.060411	0.075092
H200007656	ELF3	-	1.56	-0.64	4	0.00152	388	29.169573	0.075179
H300011756	FAM13A1	-	1.56	-0.64	4	0.001542	389	29.587307	0.07606
H300016266	BDNF	+	1.55	0.64	4	0.001547	390	29.685875	0.076118
H200003339	AVPI1	+	1.55	0.64	4	0.001548	391	29.702633	0.075966
H200007347	NTN2L	-	1.56	-0.64	4	0.001561	392	29.959624	0.076428
H300012862	CENTA1	+	1.55	0.64	4	0.001562	393	29.964223	0.076245
H300021149	CD300A	-	1.67	-0.74	3	0.001564	394	30.01406	0.076178
H300017517	HNRPH1	-	1.56	-0.64	4	0.001566	395	30.047387	0.076069
H200001454	FKBP5	+	1.55	0.63	4	0.001589	396	30.490622	0.076997
H200000430	COL7A1	-	1.56	-0.64	4	0.00159	397	30.504457	0.076837
H300017643	RRAGD	-	1.56	-0.64	4	0.001596	398	30.62837	0.076956
H200001786	ALDH1L1	+	1.55	0.63	4	0.001605	399	30.79678	0.077185
H200006638	VEGFC	+	1.55	0.63	4	0.001622	400	31.11387	0.077785
H200001435	KIAA0841	+	1.55	0.63	4	0.001622	401	31.120163	0.077606
H300020625	CD109	+	1.55	0.63	4	0.001665	402	31.940141	0.079453

H300019856	SYT7	-	1.56	-0.64	4	0.001669	403	32.017348	0.079448
H200011671	C9orf88	-	1.56	-0.64	4	0.001675	404	32.136285	0.079545
H300021463	MCTP1	+	1.55	0.63	4	0.001685	405	32.323048	0.07981
H200000760	CDO1	+	1.55	0.63	4	0.00169	406	32.434539	0.079888
H200003905	EYA2	-	1.55	-0.64	4	0.00171	407	32.81021	0.080615
H200014049	STC2	-	1.55	-0.64	4	0.001713	408	32.861587	0.080543
H200006822	PIM1	-	1.55	-0.64	4	0.00172	409	32.999813	0.080684
H300014728	SEMA3C	+	1.55	0.63	4	0.001763	410	33.829581	0.082511
H200020358	ICA1L	-	1.55	-0.63	4	0.001778	411	34.117397	0.083011
H200014641	CNN3	+	1.55	0.63	4	0.001782	412	34.196243	0.083001
H300019723	PDE4C	-	1.55	-0.63	4	0.001789	413	34.334221	0.083134
H200015613	HSPB9	-	1.55	-0.63	4	0.001851	414	35.518572	0.085794
H300008573	C9orf121	+	1.67	0.74	3	0.001862	415	35.719046	0.08607
H200003517	IRX5	+	1.54	0.63	4	0.001874	416	35.949516	0.086417
H200006395	EGFR	-	1.55	-0.63	4	0.001897	417	36.400385	0.087291
H200008074	FLCN	-	1.55	-0.63	4	0.001901	418	36.481632	0.087277
H200003318	NPL	+	1.54	0.62	4	0.001903	419	36.512707	0.087142
H200015851	KLHL24	-	1.57	-0.65	3	0.001919	420	36.820792	0.087669
H200008258	DLGAP4	-	1.55	-0.63	4	0.001926	421	36.945702	0.087757
H300010615	LGALS8	+	1.58	0.66	3	0.001933	422	37.096551	0.087907
H300000298	UNC5B	+	1.54	0.62	4	0.001942	423	37.27005	0.088109
H200012813	FAM71A	-	1.55	-0.63	4	0.001961	424	37.618131	0.088722
H300015705	SLC26A6	-	1.55	-0.63	4	0.001967	425	37.741375	0.088803
H200004008	RNF24	-	1.54	-0.63	4	0.001987	426	38.130967	0.089509
H200001330	MOXD1	-	1.68	-0.75	3	0.002048	427	39.289213	0.092012
H300019379	DTNA	-	1.54	-0.63	4	0.002063	428	39.582918	0.092483
H300022689	LOC283849	-	1.54	-0.63	4	0.002066	429	39.648934	0.092422
H300018930	TNN	-	1.57	-0.65	3	0.002085	430	40.005757	0.093037
H300015950	FGF1	+	1.54	0.62	4	0.002098	431	40.260493	0.093412
H300009712	MYLPF	+	1.54	0.62	4	0.0021	432	40.292488	0.09327
H200003942	CLSTN1	-	1.54	-0.62	4	0.00211	433	40.475751	0.093477
H200005933	PPL	+	1.53	0.62	4	0.002135	434	40.959448	0.094377
H300002446	OR8J1	+	1.53	0.62	4	0.002143	435	41.123543	0.094537
H300018436	RRAGD	-	1.54	-0.62	4	0.002156	436	41.359533	0.094861

H300014260	RORC	-	1.54	-0.62	4	0.002195	437	42.120176	0.096385
H200014367	MICALCL	+	1.53	0.62	4	0.002214	438	42.485406	0.096999
H300014093	MC1R	-	1.54	-0.62	4	0.002225	439	42.697412	0.097261
H200005120	C1orf51	-	1.54	-0.62	4	0.002231	440	42.798612	0.09727
H200002026	SLC27A2	+	1.53	0.61	4	0.002258	441	43.328504	0.098251
H200018967	CPLX2	-	1.54	-0.62	4	0.002274	442	43.62619	0.098702
H300019655	PIAS2	-	1.54	-0.62	4	0.002296	443	44.047091	0.099429
H300011396	SLC7A8	+	1.53	0.61	4	0.0023	444	44.130711	0.099393
H200020595	GPR78	+	1.53	0.61	4	0.002302	445	44.177399	0.099275
H200016513	MKNK2	-	1.54	-0.62	4	0.002307	446	44.255874	0.099228
H300020781	FOXJ1	-	1.54	-0.62	4	0.00231	447	44.328822	0.09917
H200004943	RAI2	-	1.54	-0.62	4	0.002314	448	44.396046	0.099098
H200015481	HIST1H2AL	+	1.53	0.61	4	0.002332	449	44.750206	0.099666

* (+) indicates upregulation in treated vs untreated and (-) indicates downregulation in treated vs untreated.

† Absolute fold change between untreated and treated cells

‡ Average M of biological replicates

** n = number of biological replicates

†† P = probability of obtaining the observed average M (Z-test)

‡‡ Rank = ranked genes based on obtained probabilities (i.e., the reporter with lowest P will have rank 1)

*** Expected = expected number of reporters calculated as probability times total number of reporters

‡‡‡ FDR = number of expected number of reporters divided by observed number of reporters (rank)
